# Photocatalytic Performance of Zr-Modified TS-1 Zeolites: Structural, Textural and Kinetic Studies

**DOI:** 10.3390/molecules31020209

**Published:** 2026-01-07

**Authors:** Hristina Lazarova, Borislav Barbov, Elena Tacheva, Rusi Rusew, Stela Atanasova-Vladimirova, Boris Shivachev

**Affiliations:** 1 Institute of Mineralogy and Crystallography “Acad. Ivan Kostov”-Bulgarian Academy of Sciences (IMC-BAS), Acad. G. Bonchev Str., Bl.107, 1113 Sofia, Bulgaria; barbov@imc.bas.bg (B.B.); tacheva_e@imc.bas.bg (E.T.); r.rusev93@imc.bas.bg (R.R.); 2 National Centre of Excellence Mechatronics and Clean Technologies, 8 bul. Kliment Ohridski, P.C–1756 Sofia, Bulgaria; statanasova@ipc.bas.bg; 3 Institute of Physical Chemistry, Bulgarian Academy of Sciences, 1113 Sofia, Bulgaria

**Keywords:** TS-1, TS-1 zirconium modification, photocatalysis, water purification, dye degradation, crystal violet, methylene blue, rhodamine B, methyl orange, kinetic modeling

## Abstract

TS-1 zeolite and a series of Zr-modified samples (TS-1/xZr) were synthesized and systematically characterized to investigate the influence of zirconium incorporation on structural, textural, and photocatalytic properties. The structural and textural properties of the samples were examined by XRD and nitrogen adsorption isotherms. Elemental analysis (EDXRF, SEM/EDS) and FTIR confirmed successful incorporation of Zr into the TS-1 framework. Photocatalytic tests under white light irradiation using crystal violet (CV), methylene blue (MB), rhodamine B (RhB) and methyl orange (MO) dyes revealed enhanced degradation efficiency for the Zr-containing samples, particularly TS-1/10Zr. Kinetic modeling using pseudo-first-order (PFO) and pseudo-second-order (PSO) approaches indicated that dye degradation followed mainly PSO kinetics. Reusability studies demonstrated sustained stability and recyclability of the catalysts. The improved photocatalytic performance is attributed to synergistic electronic effects between Ti and Zr species, which enhance charge separation and light absorption.

## 1. Introduction

Pollution of water bodies (lakes, rivers, oceans, aquifers or groundwater) due to human-related activities is one of the serious environmental problems [1,2]. Most of the pollution is caused by industrial factories and plants that produce or generate physical, chemical or biological substances [3]. Industrially used water must be discharged under controlled conditions, ensuring that key indicators do not exceed established water quality standards [4] and that the discharge does not have harmful effects on aquatic ecosystems or the environment [5,6]. Dyes are substances that impart color to various materials such as textiles, paper, leather, and food. They may be either natural, derived from plants, animals, or minerals, or synthetic, produced through chemical processes [7]. Dyes are used not only for aesthetic purposes but also for marking, as indicators and in medical applications [8]. Pollution and contamination of the aquatic environments with synthetic dyes poses serious environmental and health risks due to their toxicity, chemical stability, and resistance to biodegradation [9,10]. Conventional physicochemical treatments methods, such as coagulation, adsorption, and biological oxidation are often inefficient for complete dye removal or degradation and may generate secondary pollutants that require additional processing [11,12]. Consequently, advanced oxidation processes (AOPs), including photocatalysis, have emerged as promising alternatives for water remediation [13]. These processes rely on the generation of highly reactive oxygen species capable of mineralizing complex organic molecules into harmless end products such as CO_2_ and H_2_O [14]. Titanium silicalite-1 (TS-1) is a microporous zeolite with a MFI-type structure, where titanium atoms are isomorphously incorporated into the siliceous framework of MFI [15]. In TS-1 the Ti species are tetrahedrally coordinated (TiO_4_) creating isolated active sites [16] within the silica matrix. This unique structure endows TS-1 with remarkable catalytic properties, particularly in selective oxidation and photocatalytic reactions [17,18]. Moreover, TS-1 is hydrophobic, thermally stable, and exhibits molecular-sieve behavior with pore sizes around 0.54–0.56 nm, allowing the selective adsorption and transformation of small organic molecules. Its photocatalytic activity arises from ligand-to-metal charge transfer (O→Ti^4+^), but its wide band gap (~3.2 eV) restricts light absorption to the UV region. To overcome this limitation, modification of TS-1 with heteroatoms such as Fe or Nb has been explored to enhance its visible-light activity, charge separation, and overall catalytic efficiency [19]. Zirconium (Zr) is a transition metal known for its excellent thermal stability, corrosion resistance, and strong ability to form stable oxides. In catalytic materials, Zr plays a crucial role as a structural and electronic modifier. When incorporated into oxide or zeolitic frameworks, Zr^4+^ ions can alter surface acidity, enhance thermal resistance, and improve charge-carrier mobility. The presence of Zr–O–Si or Zr–O–Ti [20,21] bonds can modify the local electronic environment, narrowing the band gap and facilitating visible-light absorption. In photocatalysis, Zr acts as a promoter that suppresses electron–hole recombination and increases the lifetime of photogenerated charge carriers, leading to enhanced degradation efficiency toward dyes such as crystal violet, methylene blue, rhodamine B and methyl orange. These properties make Zr an ideal dopant or cofactor for improving the performance of Ti-based photocatalysts such as TS-1 [22,23].

Although several studies have explored the modification of Ti-based photocatalysts with zirconium, most have focused on TiO_2_–ZrO_2_ composites or post-synthetic surface deposition. Reports on the in situ isomorphous substitution of Ti^4+^ by Zr^4+^ directly within the MFI-type TS-1 framework remain scarce, and existing studies often report limited Zr incorporation, mixed-phase formation, or incomplete structural preservation.

In the present work, we present a controlled one-pot hydrothermal synthesis that enables partial replacement of Ti by Zr (Zr-modified TS-1) while maintaining the crystallographic integrity of TS-1 and achieving unusually high Ti dispersion (~8 wt%). This approach differs from conventional routes where Zr is added post-synthetically or precipitates as separate ZrO_2_ domains.

The synthesized materials (TS-1xZr, x = 5, 10, 20, 30 mol%) were systematically investigated using EDXRF, SEM/EDS, XRD, FTIR and nitrogen adsorption–desorption analyses to correlate structural and textural properties with photocatalytic activity. Furthermore, the photocatalytic behavior of the resulting materials is systematically evaluated under white-light irradiation, rather than UV, allowing assessment of the Zr–Ti synergistic effects in a broader spectral range. By combining comprehensive physicochemical characterization with kinetic modeling across four representative dyes—crystal violet (CV), methylene blue (MB), rhodamine B (RhB) and methyl orange (MO)—this study provides the first integrated structure–activity correlation for Zr-modified TS-1 zeolites, highlighting its potential application in environmentally friendly water purification technologies.

## 2. Results and Discussion

The primary objective of this study was to partially substitute Ti with Zr within the TS-1 framework in situ to enhance the photocatalytic degradation of organic dyes such as MB, MO, RhB and CV. The question was would the synthesis protocol allow the replacement of Ti with Zr during the synthesis? The synthesis route employed here differs from earlier Zr–TS-1 studies that relied on post-treatment or co-precipitation. The in situ introduction of ZrCl_4_ into the titanosilicate precursor gel leads to homogeneous substitution without detectable ZrO_2_ segregation, as confirmed by XRD and FTIR. This fine control over Zr incorporation allows evaluation of how incremental Zr loading affects crystallinity, textural parameters, and photocatalytic efficiency—relationships that have not been quantitatively addressed in prior reports.

### 2.1. Synthesis of Zirconium-Substituted TS-1 (TS-1/xZr)

**Figure 1:**
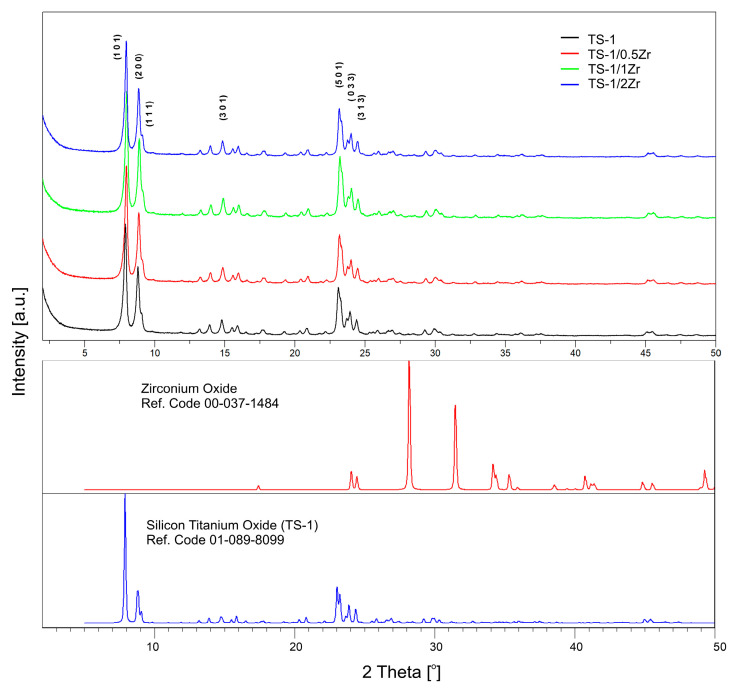
Powder X-ray diffraction (PXRD) patterns of parent TS-1 and Zr-modified samples (TS-1/0.5 Zr, TS-1/1 Zr, and TS-1/2 Zr). All samples exhibit the characteristic reflections of the MFI-type zeolite framework, confirming preservation of the TS-1 structure upon Zr modification. No reflections corresponding to crystalline ZrO_2_ phases are observed, indicating the absence of segregated zirconia domains.

The parent titanosilicate TS-1 was synthesized [24] from a viscous gel with a molar composition of 1.00 SiO_2_:0.03 TiO_2_:0.23 TPAOH:10.37 H_2_O, prepared as follows: 40 mL of tetrapropylammonium hydroxide (TPAOH) were dissolved in 10 mL of water (Solution A); 1.86 g of tetra(isopropyl) orthotitanate were mixed with 45 g of tetraethyl orthosilicate (TEOS), and the resulting mixture (Solution B) was added dropwise to the (Solution A) followed by homogenization at 60 °C for 3 h with stirring. The resulting gel was then crystallized in stainless-steel Teflon-lined autoclave under autogenous static conditions at 175 °C for 24 h. The products were (1) vacuum-filtered, (2) washed several times with distilled water, (3) dried at 60 °C for 2 h, and (4) calcinated at 550 °C for 5 h. The selected synthesis protocol allowed us to produced monophasic TS-1 with the use of TPA.OH as an organic structure-directing agent (Figure 1).

The objective, e.g., a partial substitution of Ti atoms with Zr atoms in the TS-1 framework, was performed in situ by adding 5, 10, 20 or 30 mol% ZrCl_4_ to the initial viscous gel at the expense of the same amount of TiO_2_. Thus, the molar composition of the initial gel can be given as 1.00 SiO_2_:xZrCl_4_:(0.03 − x) TiO_2_:0.23 TPAOH:10.37 H_2_O, where x = 0.0015, 0.003, 0.006 and 0.009. In order to minimize the formation of undesired crystalline phases, the temperature for the synthesis of TS-1/xZr was kept at 175 °C. The obtained products were vacuum-filtered, washed several times with distilled water, dried at 60 °C for 2 h and calcinated at 550 °C for 5 h. The above considerations resulted in the synthesis of XRD pure TS-1/xZr forms (x = 0.5, 1.02, 2.01 and 1.95 wt%).

Usually the synthesis protocols produce TS-1 with limited content of Ti, ~ 1 to 4 wt%, since the use of excessive Ti amounts may lead to the formation of extra-framework anatase or amorphous TiO_2_ phases [25]. In contrast, the present synthetic route successfully incorporated ≈8 wt% Ti and allowed its substitution by Zr while preserving the characteristic MFI structure of TS-1, as confirmed by XRD and FTIR analyses. This demonstrates that the present protocol provides superior control over Ti dispersion and framework substitution, resulting in a more efficient and optimized synthesis route compared to conventional approaches [26].

### 2.2. Physicochemical Characterization of TS-1/xZr Form

All samples of TS-1/xZr obtained were characterized by EDXRF, SEM/EDS, PXRD, FTIR and N_2_ adsorption/desorption for micro-/mesoporosity, specific surface and pore size distribution (PSD). The morphology of the materials together with elemental distributions within the Zr-modified samples were evaluated by combined SEM/TEM mapping approach. The capability of TS-1/xZr forms for photodegradation of CV, RhB, MO and MB using white light was studied.

The chemical composition of the synthesized samples was determined by EDXRF, and the results are presented in Table 1. The parent TS-1 sample contains 91.53 wt% Si and 8.47 wt% Ti. Upon Zr introduction, a slight increase in the Si wt% is observed, which reflects the normalization of elemental fractions as Ti is progressively substituted. Concurrently, the Ti content decreased from 8.47 wt% to 5.01 wt% as the Zr content increased to a maximum of 2.01 wt% for TS-1/20Zr. The inverse Ti–Zr relationship indicated partial isomorphous substitution of Ti by Zr within the TS-1 framework. Generally, the Zr incorporation follows the same trend as the nominal amount of Zr introduced in the reaction mixture. However, it is visible from the XRF data that the amount of incorporated Zr reach a saturation point in the TS-1/20Zr sample. Therefore, it is concluded that 20 mol% of Zr in the starting gel is the optimal amount for Zr incorporation. For clarity, the Zr-modified samples are denoted as TS-1/0.5 Zr, TS-1/1 Zr, and TS-1/2 Zr, where the numerical value corresponds to the wt% Zr determined by EDXRF analysis.

However, a slight decrease in Zr content is observed for TS-1/30Zr, suggesting the formation of non-framework zirconium species and indicating that the TS-1 zeolite structure has reached a saturation limit for Zr incorporation. Therefore, the TS-1/30Zr sample will not be considered in future research, as it already exhibits Zr supersaturation, and its Zr content, according to EDXRF data, is lower than that of the previous sample.

The XRD patterns of the parent TS-1 and Zr-modified samples (TS-1/0.5Zr, TS-1/1Zr and TS-1/2Zr) are shown in Figure 1. Direct comparison with the reference PXRD data of MFI-type TS-1 (ICDD pattern 01-089-8099) [27] confirms that all Zr containing materials preserve the characteristic zeolite framework. No reflections corresponding to the crystalline ZrO_2_ phase (ICDD pattern 00-037-1484) were detected, indicating the absence of isolated zirconia domains. This suggest that Zr species are either incorporated into the zeolite lattice or dispersed as X-ray amorphous species below the detection limit. The lack of distinctive Zr-related reflections is expected as Zr substitutes Ti/Si at low concentrations (up to 2.01 wt% Zr) and the newly formed Zr-O-Si linkages do not produce unique reflection but rather lead to some intensity variations or peak shifting due to difference in Zr^4+^ and Ti^4+^ ionic diameters (Zr^4+^ > Ti^4+^) [28]. Indeed, the characteristic diffraction peaks for MFI-type [29] TS-1 centered around 2θ values of 7.9°, 8.8°, 23.1°, 23.9° and 24.4° have lower intensity and minor shift in the Zr modified samples, suggesting a minor reduction in crystallinity and lattice distortion caused by incorporation of Zr-atoms in the framework.

**Figure 2:**
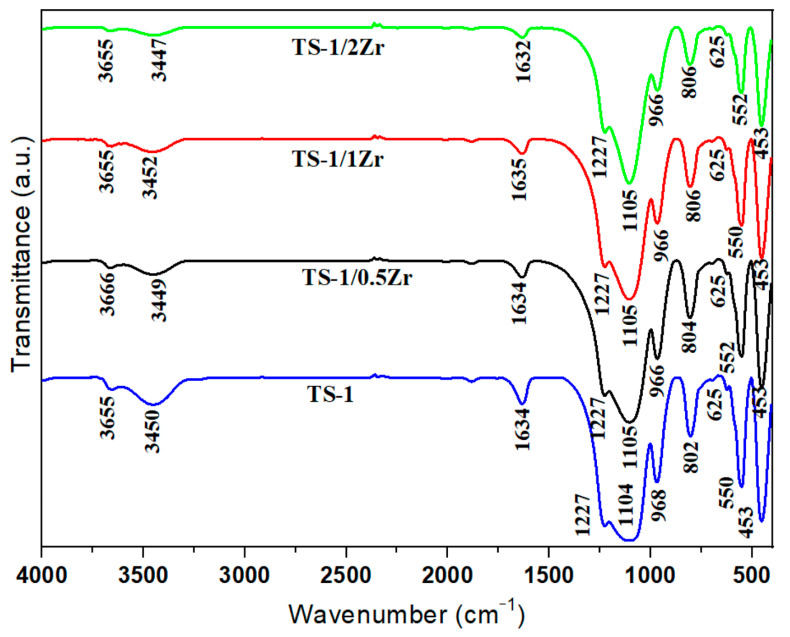
FTIR spectra of TS-1 and TS-1/xZr.

The FT-IR spectra of the TS-1 and Zr-modified TS-1 samples are presented in Figure 2. All samples exhibit typical absorption bands of the MFI-type framework at around 1227, 1105, 963, 806, and 550 cm^−1^, which are assigned to the asymmetric stretching vibrations of Si–O–Si and Si–O–Ti bonds, as well as to the double five-membered ring vibrations characteristic of the MFI structure. The persistence of these bands after Zr incorporation indicates that the zeolite framework remains intact. The broad bands at 3450–3660 cm^−1^ correspond to the stretching vibrations of surface hydroxyl groups and adsorbed water molecules, while the weak band around 1630 cm^−1^ is attributed to H–O–H bending vibrations [30,31]. The band near 960–968 cm^−1^, commonly associated with Si–O–Ti stretching vibrations, slightly shifts and decreases in intensity with increasing Zr content. Unfortunately, Zr–O–Si linkages do not have a single, universally accepted “fingerprint” band in this region that cleanly separates them from Ti-related or defect-related contributions. Based on the 960–968 cm^−1^ slight shift a partial substitution of Ti^4+^ by Zr^4+^ and possible formation of Si–O–Zr linkages into the TS-1 framework may be inferred. These results are consistent with the XRD data, further supporting that Zr is introduced into the TS-1 structure without disrupting the MFI framework.

**Figure 3:**
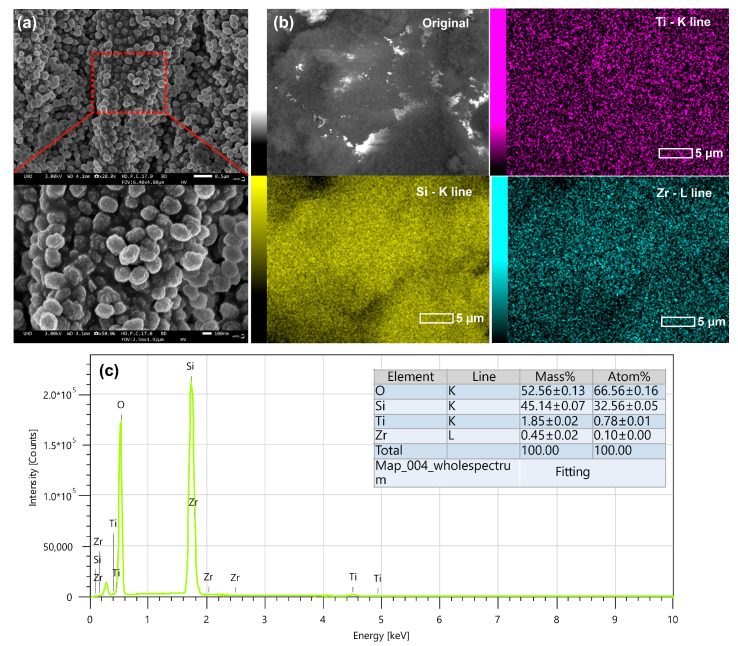
SEM image and EDS elemental mapping of the TS-1/1 Zr sample. The crystals of TS-1/1 Zr (**a**) show typical TS-1 morphology with uniform particle size. Si, Ti, and Zr are homogeneously distributed, and no Zr-rich aggregates or segregated ZrO_2_ phases are detected (**b**). EDS mapping with elemental compositions is given in table inset (**c**).

As shown in Figure 3, SEM imaging and EDS elemental mapping of the TS-1/1 Zr sample reveal well-defined TS-1 crystallites with a homogeneous spatial distribution of Si, Ti, and Zr, without detectable Zr-rich aggregates or secondary phases.

The TS-1/1 Zr sample exhibits the characteristic morphology of TS-1, consisting of submicron, well-faceted crystallites forming loosely agglomerated particles. SEM observations reveal no visible secondary phases or large Zr-rich domains. Elemental mapping reveals highly dispersed Ti and Zr species with a homogeneous spatial distribution throughout the zeolite particles, as evidenced by SEM–EDS analysis. Both Ti and Zr are uniformly distributed within the TS-1 framework, and no Zr-rich domains or detectable clustering are observed. This indicates that Zr incorporation at 1 wt% does not lead to phase segregation or the formation of distinct ZrO_2_ particles, in good agreement with the PXRD results.

The obtained UV-VIS DRS data (Tauc plots) indicate that the signal is not perfectly flat at low energies: it begins to rise gradually below the main linear absorption edge (roughly from ~4.3–4.8 eV) before the steep/near-linear region used for the band-gap extrapolation (around ~5.0 eV). The curved pre-edge region is consistent with an Urbach-type tail/defect-related sub-bandgap absorption.

Overall, the combined EDXRF, PXRD, FT-IR, UV-VIS DRS and SEM–EDS results are consistent with partial Zr incorporation into TS-1 while preserving the MFI framework, although complementary local-structure probes (e.g., XPS, XAS/EXAFS, or ^91^Zr MAS NMR) would be required for unambiguous proof of framework Si–O–Zr linkages.

**Figure 4:**
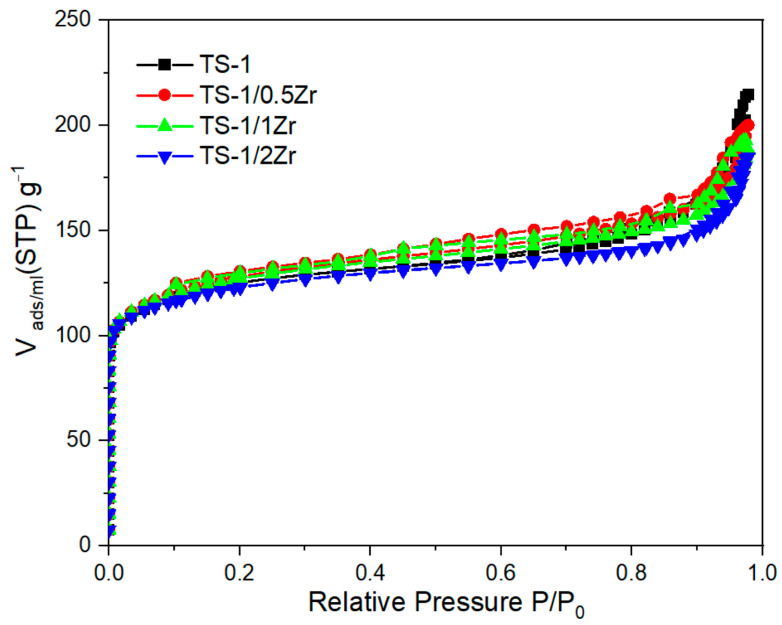
Nitrogen adsorption isotherms for TS-1 and TS-1/xZr.

The N_2_ adsorption–desorption isotherms of the TS-1 and Zr-modified samples are shown in Figure 4, and the corresponding textural parameters are summarized in Table 2. According to the IUPAC classification [32], all samples exhibit isotherms dominated by a steep uptake at very low relative pressures (P/P_0_ < 0.01), characteristic of Type I microporous materials. In addition, a weak H4-type hysteresis contribution is observed, which is commonly associated with the presence of narrow slit-like textural mesoporosity or interparticle voids. The parent TS-1 sample exhibits a specific surface area of 414.52 m^2^·g^−1^ (BET evaluated in the P/P_0_ range of 0.08–0.35), a total pore volume of 0.29 cm^3^·g^−1^, and an average pore diameter of 2.8 nm, consistent with the characteristics of microporous MFI-type zeolites. Upon zirconium incorporation, the surface area shows only minor variations, with a slight increase for TS-1/0.5Zr and TS-1/1Zr followed by a small decrease for TS-1/2Zr. Meanwhile, both the total pore volume and average pore diameter decrease marginally with increasing Zr content.

These trends suggest that low levels of Zr incorporation may slightly improve textural properties, whereas higher Zr loading can induce partial narrowing or blocking of textural pores. Overall, the relatively stable surface area and pore volume, together with the preservation of the Type I isotherm shape and H4-type hysteresis contribution, confirm that zirconium incorporation does not significantly alter the intrinsic microporous structure of TS-1.

### 2.3. Photocatalytic Degradation Efficiency

**Figure 5:**
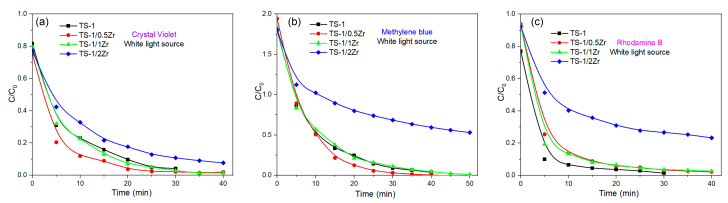
Photodegradation of (**a**) CV, (**b**) MB and (**c**) RhB promoted by white light irradiation in the presence of TS-1 (black), TS-1/0.5Zr (red), TS-1/1Zr (green) and TS-1/2Zr (blue); the line connecting the dots is provided as a guide for the eye.

Figure 5a–c and Figure S1–S4a–d present the photocatalytic degradation behavior of crystal violet (CV), methylene blue (MB), rhodamine B (RhB), and methyl orange (MO) under white light irradiation in the presence of TS-1 and Zr-modified TS-1 catalysts (TS-1/0.5Zr, TS-1/1Zr, and TS-1/2Zr). The degradation process was monitored by time-dependent UV–Vis absorption spectra and corresponding concentration decay profiles (C/C_0_), where C_0_ and C denote the initial and momentum dye concentrations, respectively. In all cases, the characteristic absorption peaks of the dyes—approximately 590 nm for CV, 663 nm for MB, 553 nm for RhB, and 498 nm for MO—decrease progressively with irradiation time, confirming successful photocatalytic degradation. The parent TS-1 catalyst exhibits measurable photocatalytic activity toward all dyes under white light; however, complete degradation requires relatively long irradiation times (30–50 min, depending on the dye). The decolorization efficiency over TS-1 follows the order MB > CV > RhB > MO, indicating dye-dependent degradation kinetics related to molecular structure and surface interactions. Incorporation of Zr into the TS-1 framework significantly accelerates the degradation process for CV, MB, and RhB. The absorbance intensity decreases more rapidly, and nearly complete decolorization is achieved within shorter irradiation times. Among the modified samples, TS-1/0.5Zr consistently demonstrates the highest photocatalytic performance, achieving almost complete degradation of CV and RhB within 30–40 min and MB within approximately 50 min. The enhanced activity is attributed to improved light absorption, more efficient charge carrier separation, and increased generation of reactive oxygen species (ROS) induced by moderate Zr incorporation. Further increasing the Zr content leads to diminished performance. The TS-1/2Zr catalyst shows a slower degradation rate for most dyes, likely due to excessive Zr causing partial pore blockage, reduced surface area, or enhanced electron–hole recombination, which limits the availability of active sites and suppresses photocatalytic efficiency. RhB degradation proceeds rapidly over all catalysts, with a sharp decrease in C/C_0_ observed within the first 10 min of irradiation. TS-1/0.5Zr and TS-1/1Zr outperform the parent TS-1 and TS-1/2Zr, achieving nearly complete degradation within 30 min, indicating that moderate Zr loading strongly enhances degradation kinetics. In contrast, MO exhibits higher resistance to photocatalytic degradation. The parent TS-1 catalyst shows slightly better performance than the Zr-modified samples, suggesting that MO interacts less effectively with the Zr-modified surface (Figure S13). This behavior is likely related to differences in molecular structure, charge distribution, and adsorption affinity, highlighting the dye-dependent nature of photocatalytic performance. Overall, the results demonstrate that controlled Zr incorporation significantly enhances the photocatalytic activity of TS-1 under white light irradiation. An optimal Zr loading (TS-1/0.5Zr) maximizes degradation efficiency by balancing light absorption, charge separation, and active site availability, while excessive Zr loading has a detrimental effect. These findings emphasize the importance of precise modification control in designing efficient photocatalysts for environmental remediation.

It should be noted that the reported photocatalytic performance refers to dye decolorization and molecular degradation, as assessed by UV–Vis spectroscopy. Complete mineralization was not evaluated in the absence of TOC analysis.

**Figure 6:**
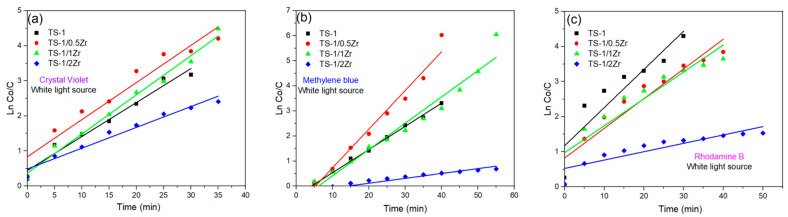
Pseudo-first-order kinetic plots (ln(C_0_/C) vs. t) for the photocatalytic degradation of (**a**) CV, (**b**) MB and (**c**) RhB over TS-1 and Zr-modified TS-1 catalysts under white light irradiation.

To quantitatively evaluate the photocatalytic degradation kinetics, the experimental data were fitted using a pseudo-first-order kinetic model, ln(C_0_/C) = k_1_t. The corresponding apparent rate constants (k_1_) and correlation coefficients (R^2^) are summarized in Table 3 and Figure 6. The results show that Zr incorporation strongly affects the degradation kinetics in a dye-dependent manner. For methylene blue, TS-1/0.5Zr exhibits the highest rate constant (k_1_ = 0.0039 min^−1^), confirming its superior photocatalytic performance. In contrast, excessive Zr loading (TS-1/2Zr) results in a significant decrease in the degradation rate for all dyes, which can be attributed to partial pore blocking and enhanced charge recombination. The relatively lower R^2^ values obtained for some systems indicate that the degradation process is not governed exclusively by first-order kinetics, consistent with the pseudo-second-order analysis discussed below.

### 2.4. Catalyst Recycling

**Figure 7:**
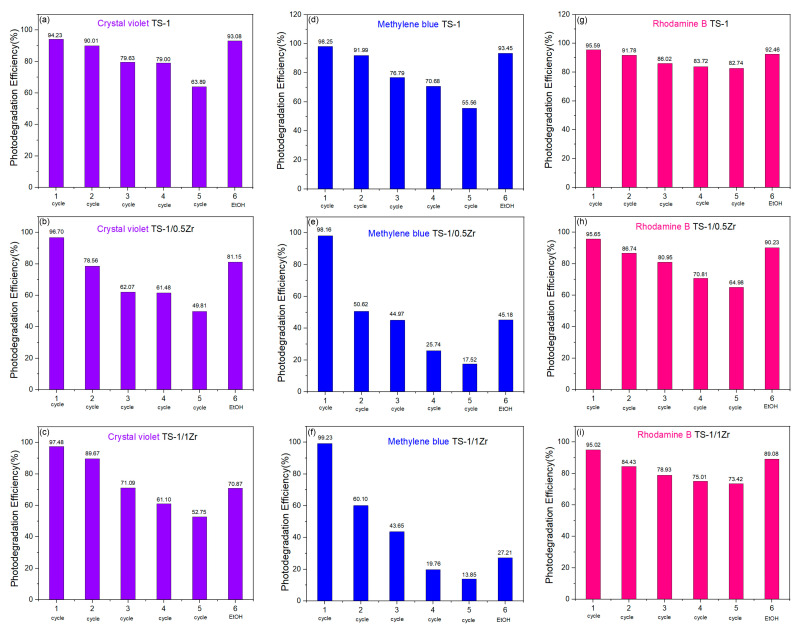
Reusability and regeneration potential of TS-1, TS-1/0.5 Zr and Ts-1/1Zr for 30 min photodegradation of (**a**–**c**) CV, (**d**–**f**) MB and (**g**–**i**) RhB; the sixth cycle discloses the catalyst recovery achieved with simple ethanol wash.

Figure 7 presents the reusability and regeneration performance of the TS-1, TS-1/0.5Zr and TS-1/1Zr photocatalysts during six successive photodegradation cycles of CV, MB and RhB under white light irradiation for 30 min per cycle. The sixth cycle corresponds to the catalyst after regeneration by a simple ethanol washing process. For all three dyes, the TS-1 photocatalyst demonstrates excellent stability and recyclability: The initial photodegradation efficiencies are high—94.2% for CV, 98.3% for MB and 95.6% for RhB. A gradual decline in the efficiency is observed after repeated use, with values dropping to about 63.9% for CV, 55.6% for MB and 82.7% for RhB by the fifth (5th) cycle. The decrease in activity over cycles can be attributed to the adsorption of dye molecules and formation of intermediates on the catalyst surface, blocking the active sites and hindering light absorption. After a “simple” ethanol washing treatment, the photodegradation efficiency is successfully restored to ~93–94% for all dyes, indicating that TS-1 maintains structural integrity and can be effectively regenerated without significant loss of performance. These results confirm that TS-1 exhibits excellent long-term stability prone to regeneration and making it a robust photocatalyst for repeated use for CV, MB or RhB dyes degradation.

The Zr-modified TS-1/0.5Zr catalyst (Figure 7b,e,h) shows similar trends but a more pronounced decrease in activity upon recycling: the initial efficiencies are slightly higher for the first cycle (96.7% for CV, 98.2% for MB, and 96.6% for RhB). However, a more significant drop in performance is observed with successive runs—reaching 49.8% for CV, 17.5% for MB, and 64.9% for RhB after the fifth cycle. The reduced reusability may result from surface deactivation or accumulation of organic residues poisoning the “catalyst”. After ethanol regeneration, the catalytic activity partially recovers (81.2% for CV, 45.2% for MB, and 90.2% for RhB), demonstrating that washing can restore some of the lost activity, but not to the full initial level.

Figure 7c,f,i illustrates the reusability and regeneration behavior of the TS-1/1Zr catalyst. A noticeable decline in photocatalytic efficiency is observed over successive cycles for all dyes. For CV, the initial degradation efficiency of 97.5% gradually decreases to 52.8% by the fifth cycle. After ethanol regeneration, the activity partially recovers to 70.9%, confirming that surface fouling contributes significantly to the deactivation and is at least partly reversible. For MB, the first cycle shows 99.2% degradation efficiency, followed by a sharp decline upon reuse, reaching only 13.9% by the fifth cycle. Ethanol washing restores the efficiency to 27.2%, indicating substantial surface deactivation, likely associated with strong dye adsorption or the accumulation of persistent intermediates. In the case of RhB, the degradation efficiency decreases from 95.0% to 73.4% over five cycles, while ethanol treatment restores activity to 89.1%, suggesting that most of the performance loss is attributed to removable surface deposits rather than irreversible structural changes. Overall, TS-1/1Zr exhibits moderate stability but undergoes progressive activity loss when reused, primarily caused by surface fouling effects. Ethanol regeneration can effectively restore a significant portion of the catalytic performance, particularly for dyes that exhibit stronger adsorption tendencies such as RhB.

The TS-1/2Zr catalyst (Figure S5) exhibits significantly lower stability and poor reusability compared to TS-1/1Zr. For CV, the initial degradation efficiency of 92.5% decreases sharply to 44.1% by the third cycle, indicating severe deactivation likely associated with excessive Zr incorporation, which can increase charge-recombination rates and introduce structural distortion. For MB, the photocatalytic efficiency declines rapidly from 73.4% in the first cycle to only 5.0% by the third cycle, reflecting an almost complete loss of activity after limited reuse. Similarly, the RhB degradation efficiency decreases from 74.8% to 13.0% over three cycles, further confirming the poor recyclability of this material. The drastic reduction in performance for TS-1/2Zr can be attributed to the high Zr loading, which may block or dilute active Ti sites, reduce the accessibility of surface-active centers, and enhance electron–hole recombination. In addition, excessive Zr incorporation may cause partial pore blockage or increased deposition of dye intermediates, resulting in heavier surface fouling that is more difficult to remove during regeneration. Indeed, the comparison of the diffractograms of the initial of the spent TS-1/0.5Zr sample after five catalytic cycles and EtOH wash (Figure S6) show that the intensity of the first two reflections (2θ ≈ 7–9°) diminishes. As peak positions remain essentially unchanged and the higher-angle peaks are still present, then the framework is largely preserved. Thus, the intensity decrease mainly indicates pore filling or partial framework damage, especially at the outer shell of crystals. The framework damage or leaching was investigated by comparing the EDS data for initial and spent samples after the 5th regeneration cycle and EtOH washing (Table 4). The wt% values for the three elements (Si, Ti and Zr) remain practically the same, and thus framework damage or leaching is not evidenced. However, we would like to emphasis that the EDS data was deliberately collected on a minimal amount of sample (100 mg) as this was the maximum available amount after five cycles and EtOH wash, no normalization effects were applied and may be subjective. For a definitive exclusion of the framework degradation and leaching a combination of methods is needed: ICP-MS/OES for the post-reaction supernatant and filtrate (before and after regeneration wash), surface-sensitive check with XPS that will compare Zr/Ti on the surface before and after cycling to see if Zr depletion is preferentially at the surface.

Overall, the reusability tests demonstrate that the long-term stability of TS-1-based photocatalysts is highly dependent on Zr content. While TS-1/0.5Zr maintains a favorable balance between activity and durability, excessive Zr incorporation (as in TS-1/1Zr and particularly TS-1/2Zr) leads to rapid deactivation and limited regeneration capability. These findings highlight the importance of controlled Zr doping to achieve efficient and reusable photocatalysts for dye degradation under white-light irradiation.

### 2.5. Kinetic Studies

The photocatalytic degradation kinetics of CV, MB, RhB and MO over the TS-1 and Zr-modified TS-1 catalysts were analyzed using the linear pseudo-first-order (PFO) kinetic model, expressed as follows [33]:

(1) $$\log\left(q_{e}-q_{t}\right)=\log q_{e}-\left(\frac{K_{1}}{2.303}\right)t$$

where C_0_ and C_t_ are the dye concentrations at initial time and time t, respectively, and k_1_ (min^−1^) is the apparent rate constant. The corresponding linear fits are presented in Figures S7–S11 for TS-1, TS-1/0.5Zr, TS-1/1Zr, and TS-1/2Zr, respectively. As shown in Figures S7–S11, the data does not fit well using a PFO model, confirming that the degradation does not follows pseudo-first-order kinetics.

**Figure 8:**
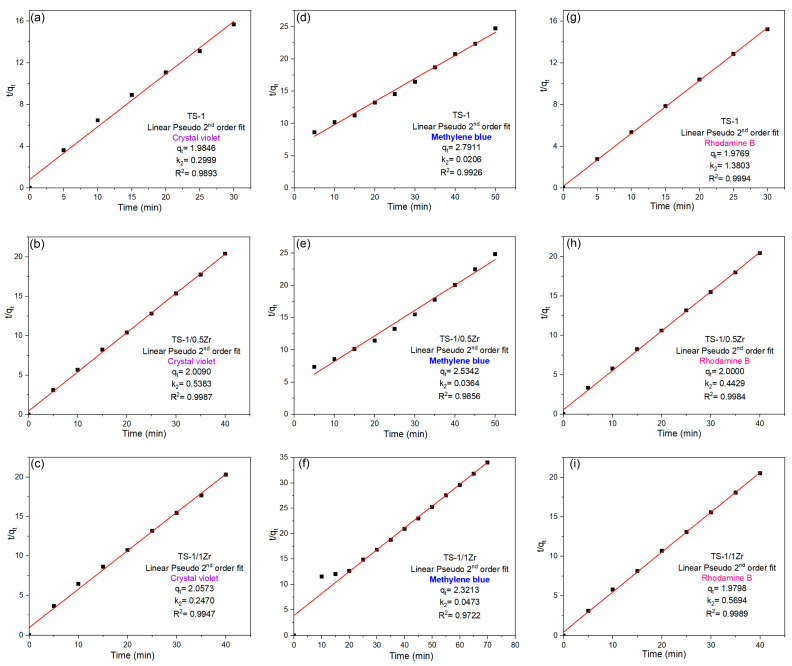
Linear PSO kinetic models fits for the adsorption of (**a**–**c**) CV, (**d**–**f**) MB and (**g**–**i**) RhB on TS-1, TS-1/0.5Zr and TS-1/1Zr.

To further evaluate the kinetics of the photocatalytic degradation process, the experimental data were fitted to the pseudo-second-order (PSO) kinetic model, expressed as follows [33,34]:

(2) $$\frac{t}{q_{t}}=\frac{1}{k_{2}{q_{e}}^{2}}+\left(\frac{1}{q_{e}}\right)t$$

where q_t_ and q_e_ (mg·g^–1^) represent the amount of dye adsorbed on the photocatalyst at time t and at equilibrium, respectively, and k_2_ (g mg^−1^ min^−1^) is the rate constant. The corresponding linear plots are displayed in Figure 8a–i for the TS-1, TS-1/0.5Zr, TS-1/1Zr, and TS-1/2Zr photocatalysts, respectively. For all dyes, the plots of t/q_t_ versus t show good linearity, confirming that the PSO model adequately describes the kinetic behavior of the TS-1 catalyst. The correlation coefficient (R^2^) values are in the range 0.972 to 0.999 and are significantly higher than those obtained for the PFO model, indicating that the photodegradation of dyes over TS-1 mainly follows pseudo-second-order kinetics. Among the dyes, methylene blue (MB) exhibits the highest reaction rate, while methyl orange (MO, Figure S12) shows the slowest degradation, consistent with their adsorption affinities. The TS-1/0.5Zr sample shows excellent linear correlation (R^2^ ≈ 0.99) with the PSO model for all dyes, suggesting that the rate-limiting step involves chemisorption or electron transfer between the catalyst surface and dye molecules. The rate constants (k_2_) are significantly higher than those for pure TS-1, confirming that moderate Zr doping enhances surface reactivity and charge-transfer efficiency. This improvement is consistent with the PSO results and indicates that both adsorption and photocatalytic oxidation steps are accelerated in the Zr-modified catalyst. For the TS-1/1Zr catalyst, the PSO kinetic plots also exhibit good linearity, though with slightly reduced slopes compared to TS-1/0.5Zr. This suggests that while Zr addition continues to improve adsorption capacity, excess Zr begins to hinder the charge separation process, decreasing the overall degradation rate. The kinetic data imply that both physical adsorption and surface-mediated reactions contribute to dye degradation, but electron–hole recombination becomes more prominent at higher Zr contents. The highest kinetic constants and best model fits are consistently observed for the TS-1/0.5Zr catalyst, confirming its superior surface activity and reaction kinetics. Moderate Zr doping effectively optimizes the balance between adsorption, charge transfer, and photocatalytic reaction rates. The pseudo-second-order kinetic analysis supports that the dye degradation over TS-1-based photocatalysts involves chemisorption and surface reaction steps. Optimal Zr content (0.5 wt%) provides the most favorable kinetics, while higher loadings (1 Zr and 2 Zr) impair catalyst performance due to structural and electronic limitations. Therefore, TS-1/0.5Zr demonstrates the best kinetic performance, aligning with the photocatalytic degradation trends observed in earlier sections.

### 2.6. Plausible Photodegradation Mechanism of MB, CV and RhB by TS-1, TS-1/0.5Zr and TS-1/1Zr

**Figure 9:**
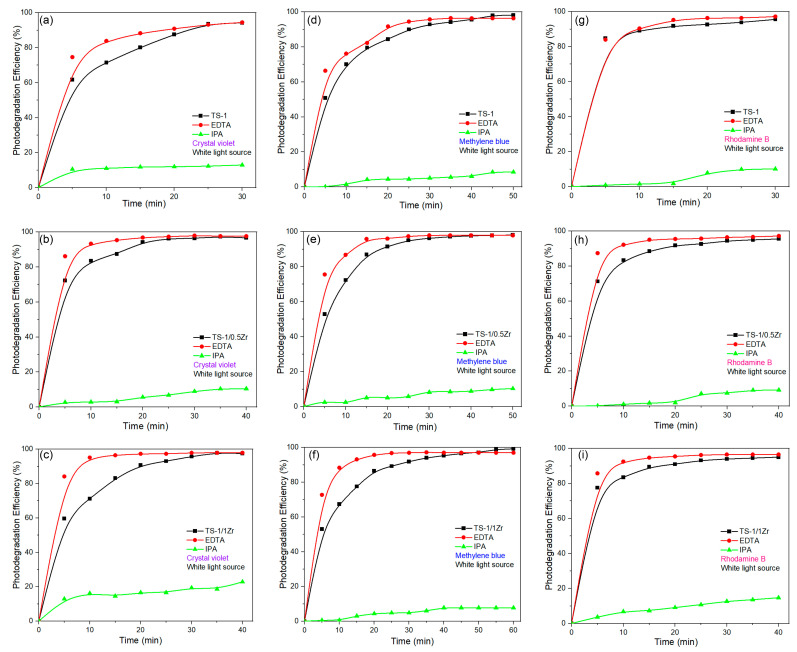
The effect of EDTA and IPA scavengers on the degradation process of (**a**–**c**) CV, (**d**–**f**) MB and (**g**–**i**) RhB by TS-1, the line connecting the dots is provided as a guide for the eye.

Figure 9 show the influence of different radical scavengers—EDTA (a hole scavenger) and isopropanol (IPA, a hydroxyl radical scavenger)—on the photocatalytic degradation of CV [35,36], MB [37,38] and RhB [39,40] under white light irradiation for TS-1, TS-1/0.5Zr and TS-1/1Zr catalysts, respectively. These experiments were conducted to determine the dominant reactive species involved in the photocatalytic process. For the pure TS-1 catalyst (Figure 9a,d,g), the addition of IPA causes a significant decrease in photodegradation efficiency for all dyes, nearly suppressing the degradation activity. In contrast, the addition of EDTA results in only a slight decrease in photocatalytic performance compared to the control (without scavenger). This indicates that hydroxyl radicals (•OH) are the main active species responsible for dye degradation in the TS-1 photocatalytic system, while photogenerated holes (h^+^) play a secondary role [41,42,43]. The strong inhibition caused by IPA suggests that the TS-1 catalyst promotes effective generation of hydroxyl radicals through surface-bound Ti–OH groups under white light excitation. For the Zr-modified catalyst TS-1/0.5Zr (Figure 9b,e,h) and TS-1/1Zr (Figure 9c,f,i), the same trend is observed—IPA drastically suppresses degradation, while EDTA has only a mild effect—confirming that •OH radicals remain the key oxidative species. However, the overall degradation rates are higher than those of TS-1, demonstrating that Zr incorporation enhances •OH radical formation efficiency. This improvement can be attributed to better charge separation and stronger surface redox activity due to the synergistic interaction between Ti and Zr centers. These findings indicate that Zr modification promotes more effective utilization of photogenerated electrons and holes, leading to higher concentrations of hydroxyl radicals and, consequently, faster dye degradation. The radical scavenger experiments confirm that, the hydroxyl radicals (•OH) are the dominant oxidizing species in both TS-1 and Zr-modified catalyst systems. The hole contribution (h^+^) is relatively minor but still assists in the initial oxidation of adsorbed dye molecules. Zr doping enhances the generation of •OH radicals by improving charge carrier separation and facilitating the activation of surface-adsorbed water or oxygen species.

The mechanism can thus be summarized as follows:

White light → TS-1/xZr → e^−^ + h^+^

h^+^ + H_2_O → •OH + H^+^

•OH + Dye→ Degraded products

The reactive species trapping experiments clearly demonstrate that hydroxyl radicals (•OH) are the main reactive species governing the photocatalytic degradation of organic dyes by TS-1-based catalysts. Zr modification significantly enhances •OH radical generation and overall photocatalytic efficiency, confirming the critical role of surface oxygen activation and charge separation improvement in the enhanced activity of the modified photocatalyst.

The improved photocatalytic activity of TS-1/0.5Zr can be attributed to the synergistic interaction between isolated Ti sites and moderately incorporated Zr^4+^ species within the zeolite framework. The introduction of small amounts of Zr modifies the local coordination environment around Ti, generating additional surface hydroxyl groups and defect sites that facilitate charge separation. This promotes more efficient generation of •OH radicals, which were identified as the dominant oxidative species in the scavenger tests. However, when the Zr content becomes excessive, as in TS-1/1Zr and TS-1/2Zr, structural distortion and partial pore blockage reduce the accessibility of active Ti centers, while increased defect density accelerates electron–hole recombination. Thus, the balance between improved surface reactivity and structural/electronic perturbation determines the overall catalytic efficiency, with 0.5 wt% Zr representing the optimal loading.

**Figure 10:**
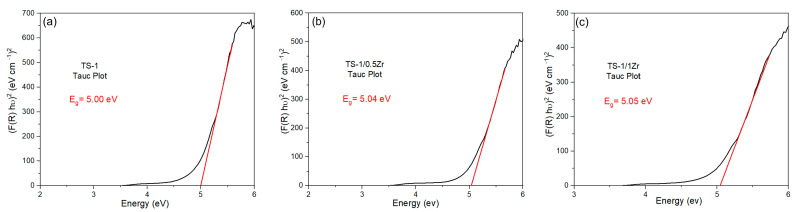
Energy band gap for (**a**) TS-1, (**b**) TS-1/0.5Zr and (**c**) TS-1/1Zr samples obtained from Tauc plots.

Diffuse Reflectance Spectroscopy (DRS) was used to evaluate the optical properties of the synthesized catalysts and to estimate their apparent band-gap energies. The band-gap values were determined from the diffuse-reflectance data using the Kubelka–Munk function, which relates the reflectance of powdered samples to their absorption characteristics [44]. Figure 10 presents the Tauc plots constructed from the UV–Vis DRS spectra of the parent TS-1 and the Zr-modified TS-1 materials. The plots of (F(R)hν)^2^ versus photon energy (hν) were used to estimate the optical band-gap (Eg) by extrapolating the linear region of the curve to the energy axis. The parent TS-1 sample (Figure 10a) exhibits an apparent band gap of 5.00 eV. Although this value appears larger than that of classical semiconductors, it is fully consistent with the optical behavior of titanium silicate materials, where the dominant transition originates from ligand-to-metal charge transfer (LMCT) between O^2−^ (2p) orbitals and isolated tetrahedral Ti^4+^ centers [45]. In zeolite frameworks such as TS-1, Ti species are highly isolated and exclusively tetrahedrally coordinated, which shifts the LMCT transition to higher energies (shorter wavelengths), resulting in an apparent band gap close to 5 eV. For the Zr-modified TS-1/0.5Zr sample (Figure 10b), the band gap increases slightly to 5.04 eV. This subtle blue shift can be attributed to the incorporation of Zr^4+^ ions into or near the TS-1 framework, which modifies the local Ti–O–Si environment and subtly influences the electronic structure around isolated Ti^4+^ sites. Despite this minor increase, TS-1/0.5Zr shows the highest photocatalytic activity, confirming that enhanced charge separation and improved surface reactivity outweigh the slight changes in light absorption. The TS-1/1Zr catalyst (Figure 10c) displays a band gap of 5.05 eV, indicating a continued, although slight, shift toward higher energy. This trend suggests increasing perturbation of the Ti–O network at higher Zr contents, which may contribute to the reduced photocatalytic efficiency observed for this sample. The TS-1/2Zr catalyst (Figure S14) exhibits the widest band gap (5.09 eV). The more pronounced blue shift likely reflects greater lattice distortion, partial blocking of Ti sites, and changes in the local electronic structure, all of which correspond with its lower photocatalytic performance and reduced reusability. It should be noted that the use of organic dyes as probe pollutants may introduce dye sensitization effects under visible light irradiation. Therefore, the observed photocatalytic activity is most reasonably attributed to an LMCT-driven mechanism inherent to TS-1-based materials, possibly accompanied by partial dye sensitization contributions. The Zr-dependent activity trends and the absence of universal enhancement for all dyes suggest that the photocatalytic process is not solely governed by dye sensitization.

Overall, the DRS results show that increasing Zr incorporation induces a gradual widening of the apparent band gap from 5.00 to 5.09 eV. This behavior is consistent with the sensitivity of LMCT transitions to local structural and electronic perturbations around isolated tetrahedral Ti centers. Importantly, despite the small variations in band gap, the superior activity of TS-1/0.5Zr demonstrates that photocatalytic performance is governed primarily by charge-carrier dynamics, surface hydroxylation, and accessibility of active Ti sites rather than by the optical absorption edge alone. Moderate Zr loading therefore provides the optimal balance between structural preservation, efficient charge separation, and photocatalytic reactivity.

The optical band gap energies (Eg), estimated from Tauc plots, are 5.00 ± 0.04 eV for TS-1, 5.04 ± 0.04 eV for TS-1/0.5Zr, 5.05 ± 0.03 eV for TS-1/1Zr, and 5.09 ± 0.04 eV for TS-1/2Zr. The observed differences between the band gap values are small and overlap within the experimental uncertainty, indicating that Zr incorporation does not induce significant band gap modification. Therefore, the enhanced photocatalytic performance of Zr-modified TS-1 cannot be attributed to band gap narrowing but is more likely related to structural and surface-related effects influencing charge carrier utilization and reactive oxygen species generation.

For comparison, the photodegradation efficiencies of other catalysts toward MB, CV, RhB and MO taken from the literature are provided in Table 5. The values of the reported efficiencies of TS-1/xZr compete with the most promising candidates.

## 3. Materials and Methods

All starting reagents for the synthesis of TS-1and TS-1/xZr forms and those used for photocatalytic activity experiments were purchased from Sigma Aldrich (Schnelldorf, Germany) or Thermo Fisher Scientific (Waltham, MA, USA) and used without additional purification. The reagents used included tetraethyl orthosilicate (TEOS, 98%, Sigma Aldrich), tetrapropylammonium hydroxide (TPAOH, 25% in water, Thermo Fisher Scientific), titanium (IV) isopropoxide (C_12_H_28_O_4_Ti, 98+%, Thermo Fisher Scientific), Zirconium (IV) chloride (ZrCl_4_, 99.9%, Sigma Aldrich), methylene blue hydrate (>97%, Sigma Aldrich), crystal violet (97%, Sigma Aldrich), methyl orange (85%, Sigma Aldrich) and rhodamine B (for fluorescence, Sigma Aldrich). The water used was ultrapure water (electrical conductivity—0.055 µS/cm, Q-FRONT EDI/BIO, Adrona, Riga, Latvia)

Elemental composition was determined using an Epsilon-1 energy-dispersive X-ray fluorescence (EDXRF) spectrometer (Malvern Panalytical, Amelo, The Netherlands) equipped with Omnian software for standardless quantification (version 2.3.B/2022). Samples were finely ground and placed in holder in the form of loose powder. Measurements were performed under ambient conditions using silver (Ag) anode X-ray tube. The instrument’s software automatically optimized the operating voltage and current based on the elements of interest.

Powder X-ray diffraction analysis was performed on Empyrean diffractometer equipped with a PIXcel3D detector and copper X-ray source (Cu*Ka* = 1.5406 Å) (Malvern Panalytical, Almelo, The Netherlands). The diffraction patterns were collected in the 3–70° 2Theta range, under operating conditions of 40 kV and 30 mA and a step size of 0.013°.

The specific surface area and porosity of TS-1 and TS-1/xZr samples were analyzed using 3Flex surface analyzer (Micromeritics, Norcross, GA, USA). Before analysis, the samples were degassed in situ at 90 °C for 5 h under vacuum (>1.10^−^^6^ mmHg). The physisorption experiments were carried out under liquid nitrogen (77 K) using N_2_ probe molecule. Quantitative information for the specific surface area (S, m^2^·g^–1^), micropore volume (V_m_, cm^3^·g^–1^), pore size distribution, etc., was obtained by analyzing the resulting N_2_ adsorption/desorption isotherms. Brunauer, Emmett and Teller (BET) specific surface areas were calculated from adsorption data in the relative pressure range from 0.05 to 0.31 P/P_0_ [57]. The total pore volume was estimated based on the amount adsorbed at a relative pressure of 0.96 [32]. Micropore volumes were determined using the *t*-plot method [58] and the Horvath–Kawazoe micropore algorithm [59]. Pore size distributions (PSDs) were calculated from nitrogen adsorption data using an algorithm based on the ideas of Barrett, Joyner and Halenda (BJH) [60]. The mesopore diameters were determined as the maxima on the PSD for the given samples.

The samples were examined using a Tensor 37 (Bruker, Berlin, Germany) spectrometer using KBr pellets. For each sample, 128 scans were collected at a resolution of 2 cm^–1^ over the wavenumber region 4000–400 cm^–1^.

Images and SEM-EDS analyses were collected on a JEOL IT800SHL (Jeol Ltd., Tokyo, Japan). The two EDS detectors, (JED 2300, Jeol Ltd., Tokyo, Japan) 100 mm^2^ providing the possibility for high-sensitivity EDS analysis and acceleration voltage of 10 kV.

The effective photocatalytic degradation of the organic dyes—CV, MB, RhB and MO—in the presence of Zr-substituted TS-1 was evaluated using UV-Vis spectroscopy. The details of the experiments were as follows: 50 mg of zeolite (TS-1/xZr) was added to 10 mL of a 10 µg/mL CV, MB, MO or RhB aqueous solution. The solution was continuously stirred for up to 70 min at ambient conditions. The absorption resulting from the photodegradation of the dye was monitored at 585 nm for CV, 663 nm for MB, 464 nm for MO and 553 nm RhB using DRAWELL spectrophotometer (Yuzhong District, Chongqing, China) or Cary 4000 (Agilent, Santa Clara, CA, USA). The kinetics of the photodegradation reactions were examined based on the change in dye concentration by measuring the characteristic absorbance peak at different irradiation times. The efficiency of photodegradation was determined using the following equation [61,62]:

(3) $$Degradation\;efficiency(\%)=\frac{C_{0}-C}{C_{0}}$$

where C_0_ and C are the solution concentration at t = 0 and after some irradiation time.

The synthesis yield was not quantitatively determined, as the primary focus of this work was on controlled framework modification and photocatalytic performance rather than process optimization. All samples were prepared under identical conditions and showed comparable solid recovery after hydrothermal crystallization and calcination, indicating good reproducibility of the synthesis procedure. The synthesis follows a conventional TS-1 hydrothermal route, and the overall preparation time is comparable to that reported for TS-1-based materials in the literature.

## 4. Conclusions

In this work, a series of Zr-modified TS-1 catalysts (TS-1, TS-1/0.5Zr, TS-1/1Zr and TS-1/2Zr) were synthesized through a controlled in situ hydrothermal approach and evaluated for the photocatalytic degradation of crystal violet, methylene blue, rhodamine B and methyl orange under white-light irradiation. The influence of Zr incorporation on structural, optical and photocatalytic properties was systematically assessed. All samples demonstrated photocatalytic activity, with the degradation efficiency strongly dependent on Zr loading.

Among the prepared catalysts, TS-1/0.5Zr exhibited the highest activity, achieving rapid decolorization for most dyes. This enhancement is likely associated with more efficient utilization of photogenerated charge carriers and enhanced reactive oxygen species generation, as inferred from the photocatalytic kinetics and scavenger experiment, increased surface reactivity and optimal dispersion of Zr within the TS-1 framework. Higher Zr contents (TS-1/1Zr and TS-1/2Zr) led to reduced activity due to framework distortion, increased electron–hole recombination and partial blocking of active sites. Kinetic analysis showed that the degradation processes followed predominantly pseudo-second-order behavior, indicating the involvement of surface-mediated chemisorption and electron-transfer processes.

Reusability experiments confirmed that TS-1 and TS-1/0.5Zr maintain good structural stability over several cycles, whereas catalysts with higher Zr loadings exhibited more pronounced deactivation, likely caused by blockage or decrease active Ti sites, lower amounts of accessible active centers or irreversible surface fouling. Radical-scavenger tests identified hydroxyl radicals (•OH) as the primary reactive species responsible for dye degradation. Diffuse reflectance optical measurements revealed a slight increase in band-gap energy with increasing Zr content; however, this effect did not negatively impact the performance of the optimally doped material.

Overall, the results show that moderate Zr incorporation (~0.5 wt%) provides the most favorable balance between charge-carrier separation and framework stability, leading to superior photocatalytic activity under white-light conditions. Unlike earlier studies on Zr-modified TS-1—typically restricted to UV excitation or single-dye testing—this work offers a comprehensive correlation between Zr content, optical behavior, kinetic performance and recyclability. The novelty of this study lies in the controlled in situ introduction of framework-integrated Zr–Ti centers and the multi-parameter evaluation that collectively identifies an optimal Zr-loading regime for sustainable water-purification applications.

## Figures and Tables

**Table 1:** EDXRF—Elemental analysis of the synthesized TS-1/xZr samples.

Samples	Si, wt%	Ti, wt%	Zr, wt%
TS-1	91.53	8.47	-
TS-1/5Zr	91.37	8.20	0.5
TS-1/10Zr	91.89	7.10	1.02
TS-1/20Zr	92.38	5.60	2.01
TS-1/30Zr	93.04	5.01	1.95

**Table 2:** Textural parameters for TS-1 and TS-1/xZr as deduced from Nitrogen adsorption isotherms.

Samples	S_BET_ m^2^·g^–1^	V_t_ cm^3^·g^–1^	Average Pore Diameter nm
TS-1	414.52	0.29	2.8
TS-1/0.5Zr	425.40	0.27	2.7
TS-1/1Zr	421.65	0.26	2.6
TS-1/2Zr	405.80	0.25	2.6

**Table 3:** Apparent pseudo-first-order rate constants (k_1_) and correlation coefficients (R^2^) for the photocatalytic degradation of CV, MB and RhB under white-light irradiation.

Catalyst	Crystal Violet		Methylene Blue		Rhodamine B	
	k_1_ (min^−1^)	R^2^	k_1_ (min^−1^)	R^2^	k_1_ (min^−1^)	R^2^
TS-1	0.0032	0.9649	0.0023	0.9920	0.0036	0.8028
TS-1/0.5Zr	0.0026	0.9320	0.0039	0.9771	0.0021	0.8934
TS-1/1Zr	0.0028	0.9846	0.0019	0.9608	0.0019	0.8396
TS-1/2Zr	0.0015	0.9635	0.0004	0.8835	0.0005	0.7981

**Table 4:** EDXRF—Elemental analysis of the modified TS-1/xZr samples after 5th regeneration cycle and EtOH washing.

Samples	Si, wt%	Ti, wt%	Zr, wt%
TS-1/0.5Zr	90.98	8.71	0.31
TS-1/0.5Zr-EtOH-MB	91.13	8.66	0.22
TS-1/0.5Zr-EtOH-RhB	90.86	8.90	0.24
TS-1/1Zr	91.80	7.66	0.54
TS-1/1Zr-EtOH-MB	91.76	7.73	0.51
TS-1/1Zr-EtOH-RhB	92.11	7.57	0.42

**Table 5:** Comparative degradation efficiencies of various catalysts toward CV, MB, MO and RhB.

Catalyst	Degradation Efficiency (%)				Ref.
	CV	MB	RhB	MO	
TS-1/C_3_N_4_-A			91.0		[39]
TS-1/C_3_N_4_-B			97.0		[39]
TS-1/C_3_N_4_-C			70.0		[39]
Ag-OMS	100.0				[46]
Bi_2_O_3_-NiO				100.0	[47]
Co-doped-NiO	98.4		95.2		[40]
TiO_2_-ZI		99.4			[48]
TiO_2_-ZS		97.3			[48]
TiO_2_/ANA	95.3				[49]
TiO_2_/CAN	91.6				[49]
SnP/AA@TiO_2_			95.0		[50]
Ag_3_PO_4_/TiO_2_-SiO_2_			100.0		[51]
rGO-ZnO-TiO_2_	89.6				[52]
Transition metal doped TiO_2_		99.6			[53]
Hierarchical MO-TS-1 zeolite		98.5			[45]
Hierarchically porous TS-1/modified-diatomite		96.8			[54]
PANi coated TiO_2_/SiO_2_				87.0	[55]
SLS/DG-TS		94.4			[56]
Hierarchical TS-1					[45]
FePc-TS-1		80.0			[45]
BA-TS-1		83.5			[45]
MO-TS-1		98.5			[45]
TS-1	94.2	98.3	95.6	40.5	This work
TS-1/0.5Zr	96.7	98.2	95.7	41.2	This work
TS-1/1Zr	97.5	99.2	95.0	45.2	This work
TS-1/2Zr	92.5	73.4	74.8	15.7	This work

## Data Availability

The original contributions presented in this study are included in the article/Supplementary Materials. Further inquiries can be directed to the corresponding authors.

## Supplementary Materials

The following supporting information can be downloaded at: https://www.mdpi.com/article/10.3390/molecules31020209/s1, Figures S1–S4: UV–Vis analysis of the photodegradation processes of (**a**) crystal violet (CV), (**b**) methylene blue (MB), (**c**) rhodamine B (RhB), and (**d**) methyl orange (MO) promoted by TS-1, TS-1/0.5Zr, TS-1/1Zr, and TS-1/2Zr, respectively, under white light irradiation. Figure S5: Reusability and regeneration potential of TS-1/2Zr for 30 min photodegradation of (**a**) CV, (**b**) MB, and (**c**) RhB; the sixth cycle demonstrates catalyst recovery after simple ethanol washing. Figure S6: PXRD patterns of spent TS-1/0.5Zr and TS-1/1Zr catalysts used in the photocatalytic degradation of methylene blue and rhodamine B; patterns recorded after the fifth regeneration cycle and ethanol washing are shown in blue, while the fresh samples are shown in red. Figures S7–S10: Linear pseudo-first-order (PFO) kinetic model fits for the adsorption of (**a**) CV, (**b**) MB, (**c**) RhB, and (**d**) MO on TS-1, TS-1/0.5Zr, TS-1/1Zr, and TS-1/2Zr, respectively. Figures S11 and S12: Linear pseudo-second-order (PSO) kinetic model fits for the adsorption of (**a**) CV, (**b**) MB, (**c**) RhB, and (**d**) MO on TS-1/2Zr (Figure S11) and for methyl orange adsorption on (**a**) TS-1, (**b**) TS-1/0.5Zr, and (**c**) TS-1/1Zr (Figure S12). Figure S13: Photodegradation of methyl orange under white light irradiation in the presence of TS-1 (black), TS-1/0.5Zr (red), TS-1/1Zr (green), and TS-1/2Zr (blue); lines are provided as guides to the eye. Figure S14: Energy band gap of TS-1/2Zr samples determined from Tauc plots. File S1: Excel table with the raw data from Diffuse Reflectance Spectroscopy (DRS) analysis.

## Abbreviations

The following abbreviations are used in this manuscript:

CV	Crystal violet
MB	Methylene blue
RhB	Rhodamine B
MO	Methyl orange
PFO	Pseudo-first-order
PSO	Pseudo-second-order
