# Supplementary material for: Photocatalytic Performance of Zr-Modified TS-1 Zeolites: Structural, Textural and Kinetic Studies
Source: Molecules. 2026 Jan 7;31(2):209. doi: 10.3390/molecules31020209 (PMC12843844; doi:10.3390/molecules31020209)
Supplement: Supplementary file 1 [file molecules-31-00209-s001.zip › ESI_HIL-vs2.docx]

**Supplementary Materials**

Photocatalytic Performance of Zr-Modified TS-1 Zeolites:
Structural, Textural and Kinetic Studies

Hristina Lazarova ^1,^*, Borislav Barbov ^1^, Elena Tacheva ^1,2^, Rusi Rusew ^1^, Stela Atanasova-Vladimirova ^2,3^ and Boris Shivachev ^1,^*

^1^ Institute of Mineralogy and Crystallography “Acad. Ivan Kostov”- Bulgarian Academy of Sciences
(IMC-BAS), Acad. G. Bonchev Str., Bl.107, 1113 Sofia, Bulgaria; barbov@imc.bas.bg (B.B.);
tacheva_e@imc.bas.bg (E.T.); r.rusev93@imc.bas.bg (R.R.)

^2^ National Centre of Excellence Mechatronics and Clean Technologies, 8 bul. Kliment Ohridski,
P.C–1756 Sofia, Bulgaria; statanasova@ipc.bas.bg

^3^ Institute of Physical Chemistry, Bulgarian Academy of Sciences, 1113 Sofia, Bulgaria

***** Correspondence: lazarova@imc.bas.bg (H.L.); bls@clmc.bas.bg (B.S.)

Contents

[Figure S1. UV-Vis analysis of the photodegradation processes of (a) CV, (b) MB, (c) RhB and (d) MO promoted by TS-1 and white light irradiation. 3](#_Toc217908428)

[Figure S2. UV-Vis analysis of the photodegradation processes of (a) CV, (b) MB, (c) RhB and (d) MO promoted by TS-1/0.5Zr and white light irradiation. 4](#_Toc217908429)

[Figure S3. UV-Vis analysis of the photodegradation processes of (a) CV, (b) MB, (c) RhB and (d) MO promoted by TS-1/1Zr and white light irradiation. 5](#_Toc217908430)

[Figure S4. UV-Vis analysis of the photodegradation processes of (a) CV, (b) MB, (c) RhB and (d) MO pro-moted by TS-1/2Zr and white light irradiation. 6](#_Toc217908431)

[Figure S5. Reusability and regeneration potential of TS-1/2Zr for 30 min photodegradation of (a) CV, (b) MB and (c) RhB; the sixth cycle discloses the catalyst recovery achieved with simple ethanol wash. 7](#_Toc217908432)

[Figure S6. PXRD of spent catalysts – TS-1/0.5Zr and TS-1/1.0 Zr used in the photocatalytic degradation reaction of Methylene blue and Rhodamine B. The PXRDs in blue represent the catalysts after 5th-regeneration cycles and EtOH wash. Starting material is given in Red. 7](#_Toc217908433)

[Figure S7. Linear pseudo-first-order (PFO) kinetic model fits for the adsorption of (a) crystal violet (CV), (b) methylene blue (MB), (c) rhodamine B (RhB), and (d) methyl orange (MO) on TS-1. 8](#_Toc217908434)

[Figure S8. Linear pseudo-first-order (PFO) kinetic model fits for the adsorption of (a) crystal violet (CV), (b) methylene blue (MB), (c) rhodamine B (RhB), and (d) methyl orange (MO) on TS-1/0.5Zr. 9](#_Toc217908435)

[Figure S9. Linear pseudo-first-order (PFO) kinetic model fits for the adsorption of (a) crystal violet (CV), (b) methylene blue (MB), (c) rhodamine B (RhB), and (d) methyl orange (MO) on TS-1/1Zr. 10](#_Toc217908436)

[Figure S10. Linear pseudo-first-order (PFO) kinetic model fits for the adsorption of (a) crystal violet (CV), (b) methylene blue (MB), (c) rhodamine B (RhB), and (d) methyl orange (MO) on TS-1/2Zr. 11](#_Toc217908437)

[Figure S11. Linear pseudo-second-order (PSO) kinetic model fits for the adsorption of (a) crystal violet (CV), (b) methylene blue (MB), (c) rhodamine B (RhB), and (d) methyl orange (MO) on TS-1/2Zr. 12](#_Toc217908438)

[Figure S12. Linear pseudo-second-order (PSO) kinetic model fits for the adsorption of methyl orange (MO) on (a) TS-1, (b) TS-1/0.5Zr, (c) TS-1/1Zr. 12](#_Toc217908439)

[Figure S13. Photodegradation of MO promoted by white light irradiation in the presence of TS-1 (black), TS-1/0.5Zr (red), TS-1/1Zr (green) and TS-1/2Zr (blue); the line connecting the dots is provided as a guide for the eye. 13](#_Toc217908440)

[Figure S14**.** Energy band gap for TS-1/2Zr samples obtained from Tauc plots. 13](#_Toc217908441)


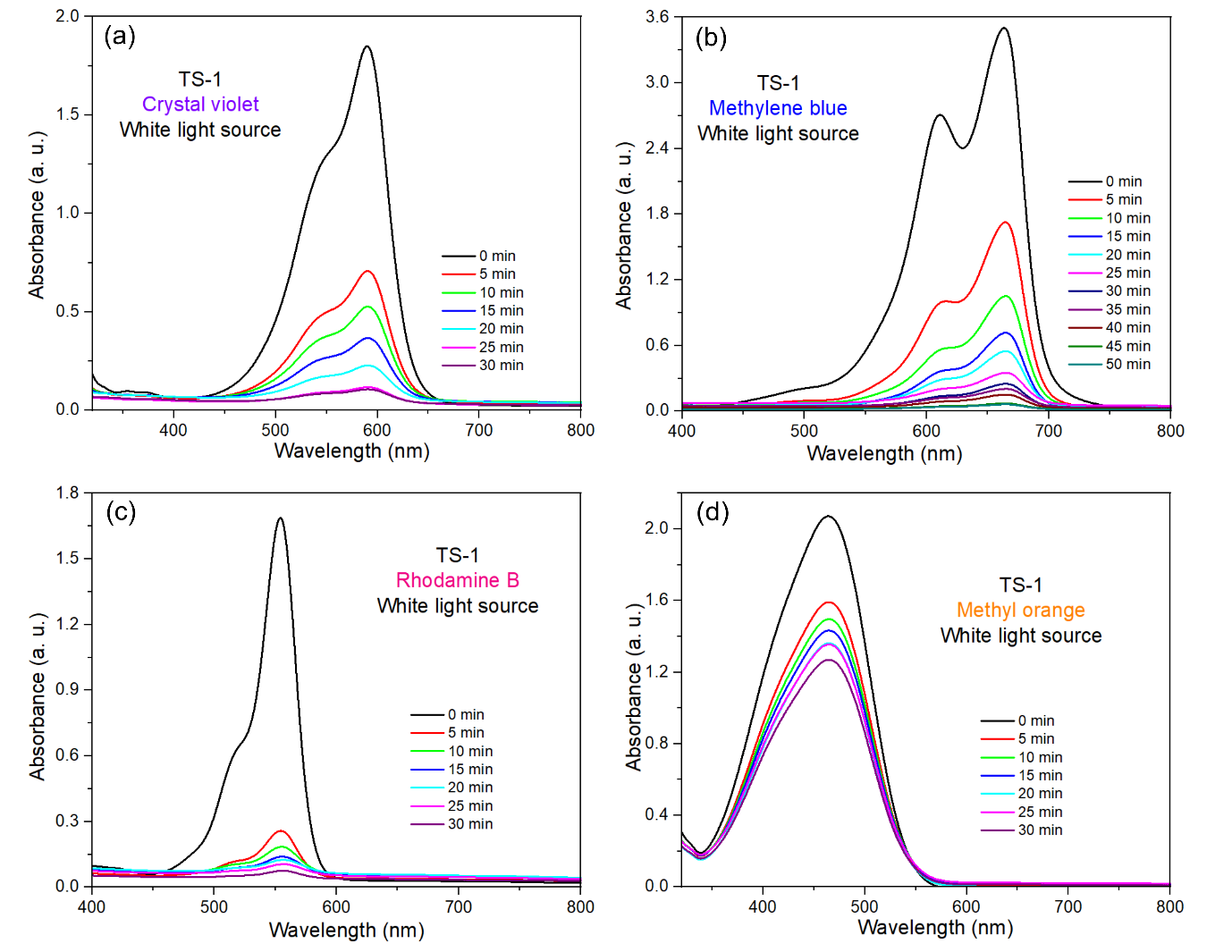


# Figure S1. UV-Vis analysis of the photodegradation processes of (a) CV, (b) MB, (c) RhB and (d) MO promoted by TS-1 and white light irradiation.


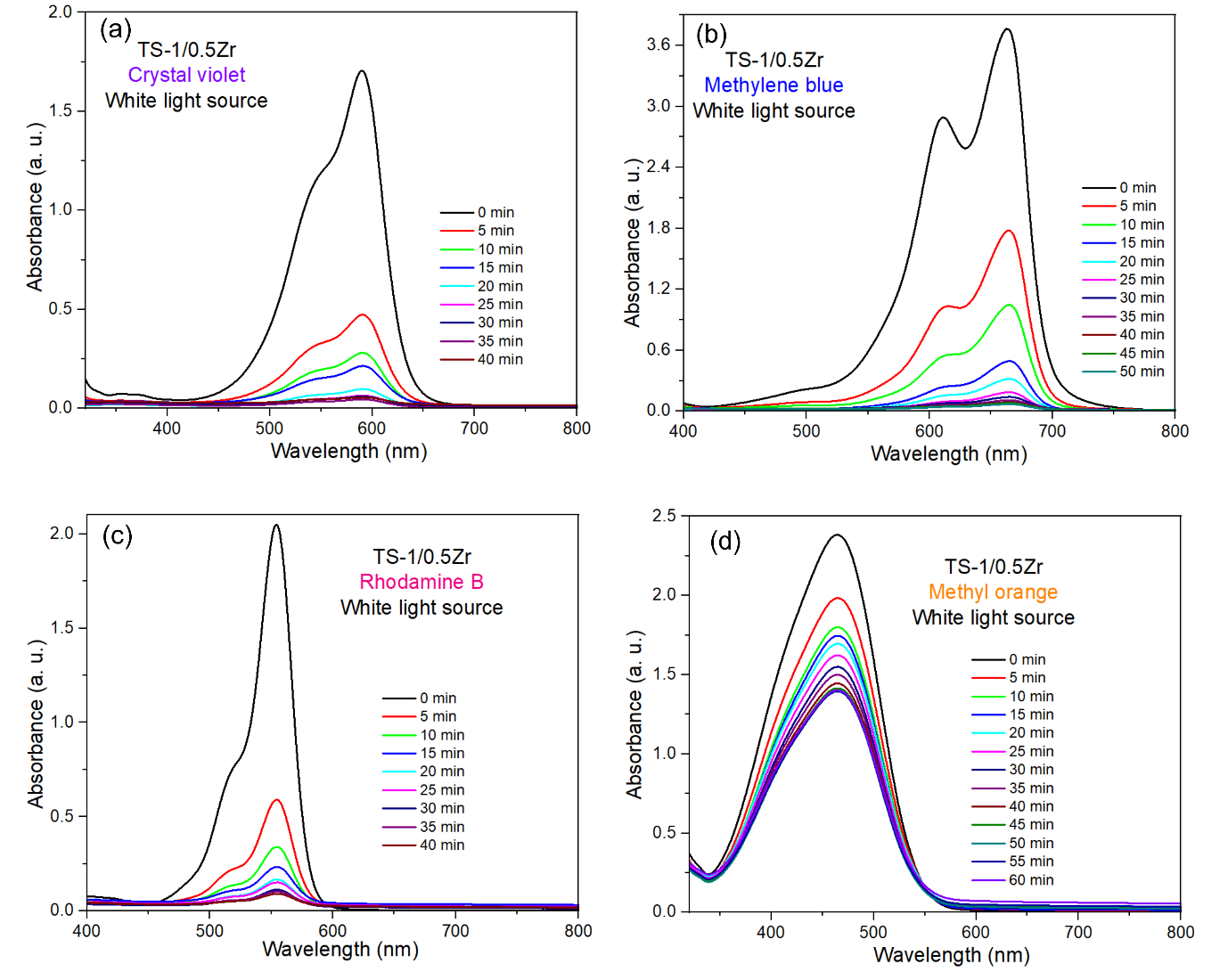


# Figure S2. UV-Vis analysis of the photodegradation processes of (a) CV, (b) MB, (c) RhB and (d) MO promoted by TS-1/0.5Zr and white light irradiation.


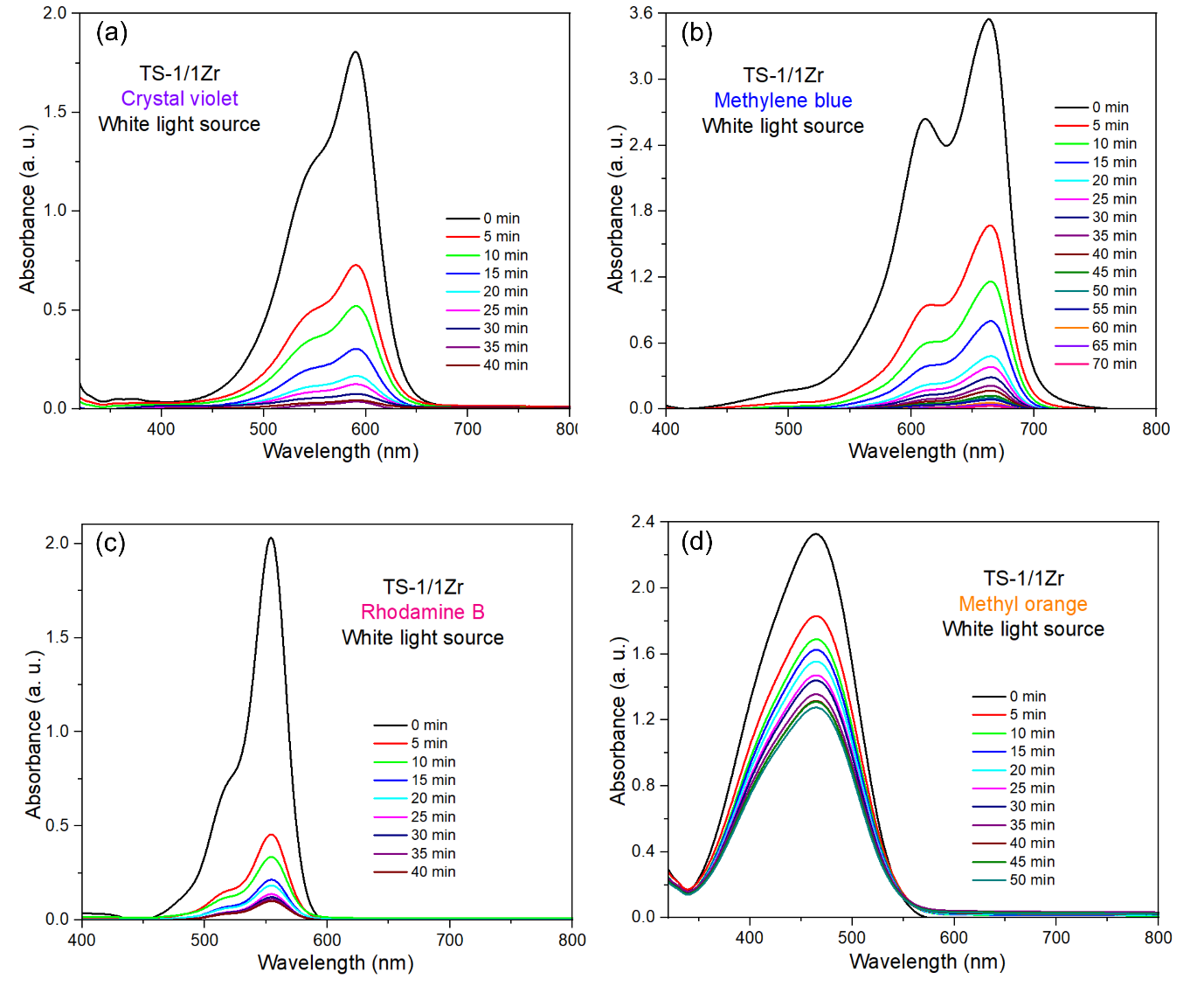


# Figure S3. UV-Vis analysis of the photodegradation processes of (a) CV, (b) MB, (c) RhB and (d) MO promoted by TS-1/1Zr and white light irradiation.


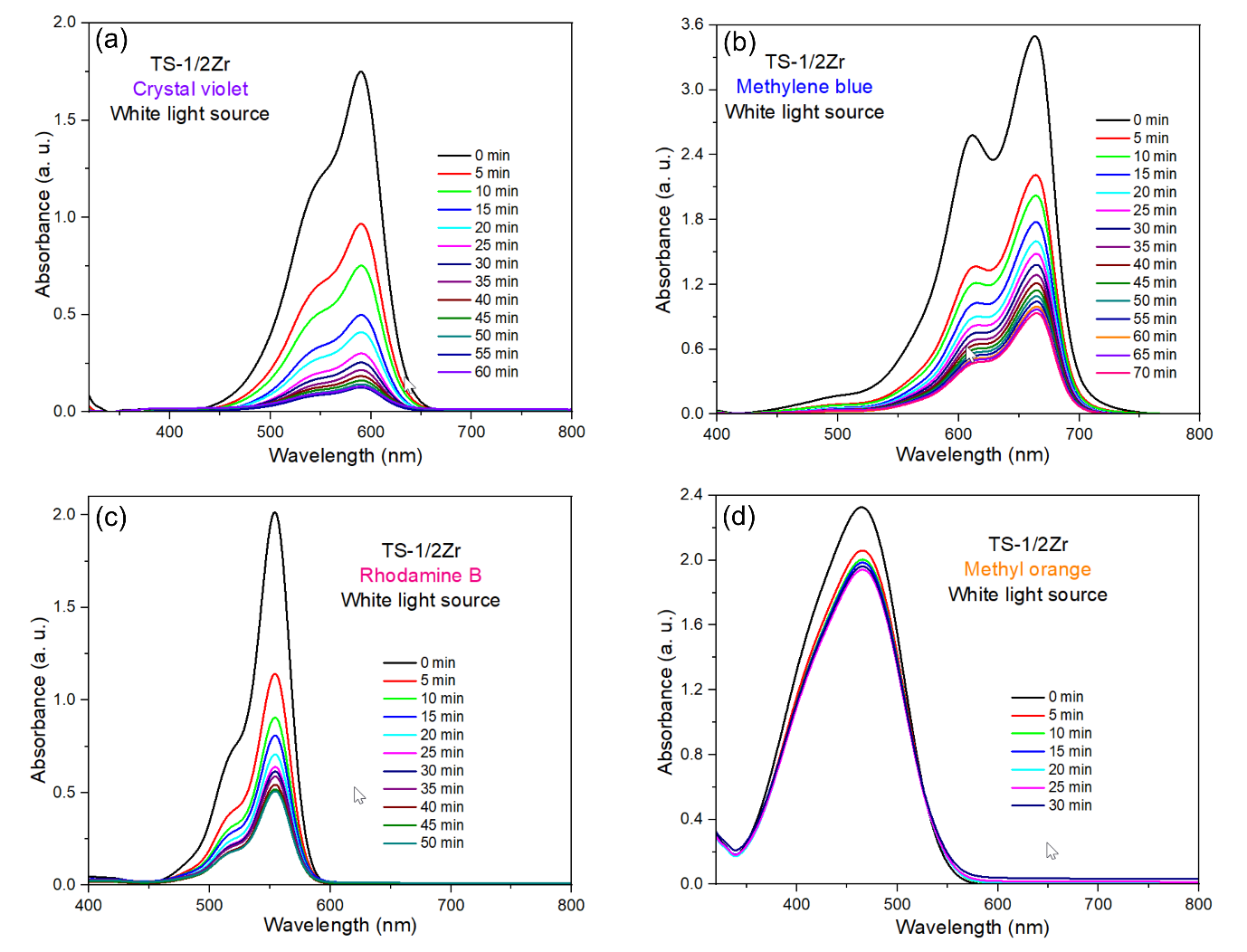


# Figure S4. UV-Vis analysis of the photodegradation processes of (a) CV, (b) MB, (c) RhB and (d) MO pro-moted by TS-1/2Zr and white light irradiation.


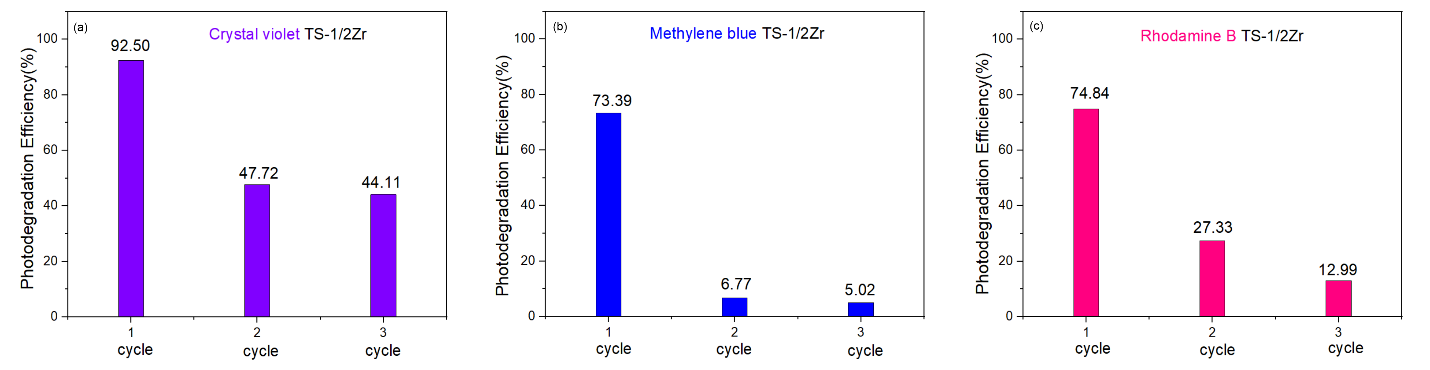


# Figure S5. Reusability and regeneration potential of TS-1/2Zr for 30 min photodegradation of (a) CV, (b) MB and (c) RhB; the sixth cycle discloses the catalyst recovery achieved with simple ethanol wash.


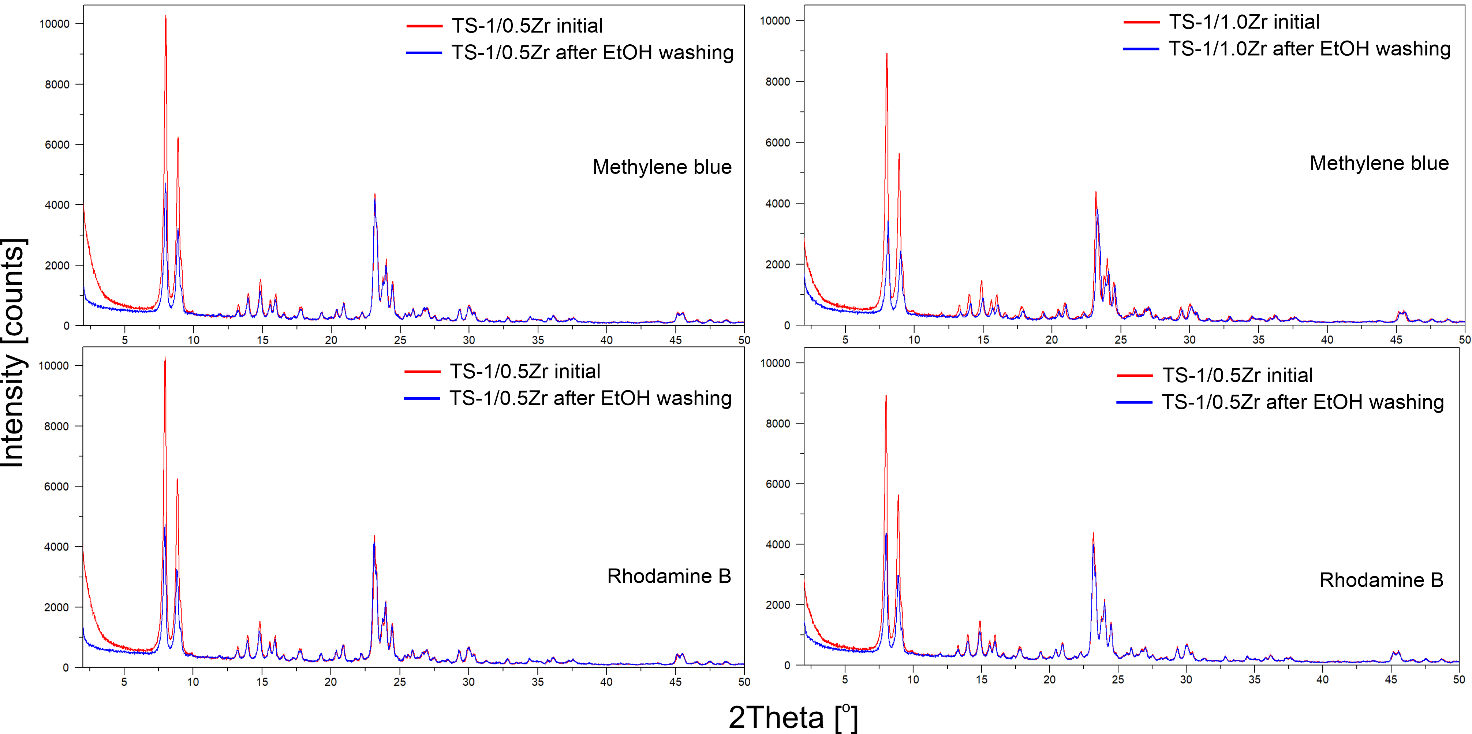


# Figure S6. PXRD of spent catalysts – TS-1/0.5Zr and TS-1/1.0 Zr used in the photocatalytic degradation reaction of Methylene blue and Rhodamine B. The PXRDs in blue represent the catalysts after 5th-regeneration cycles and EtOH wash. Starting material is given in Red.


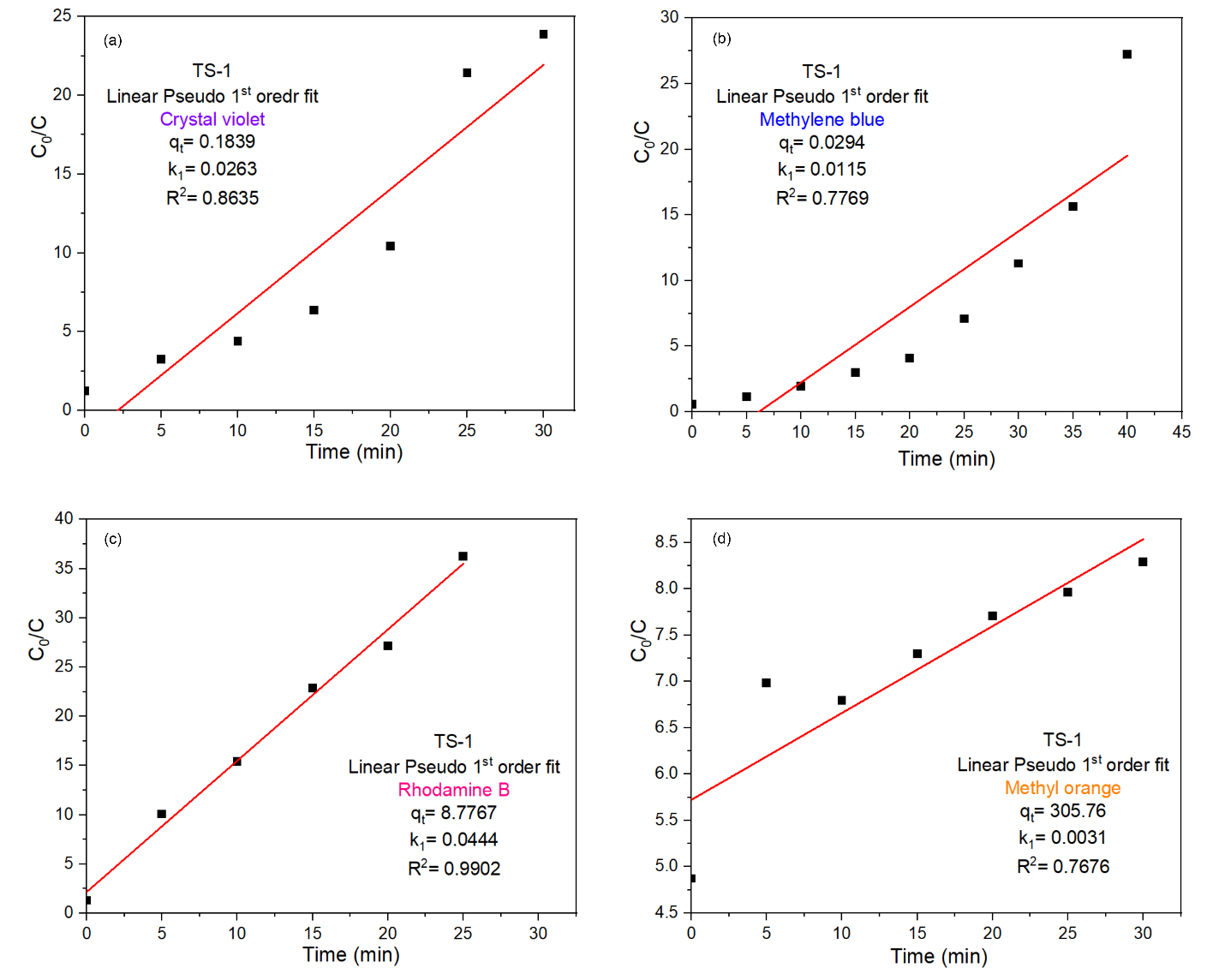


# Figure S7. Linear pseudo-first-order (PFO) kinetic model fits for the adsorption of (a) crystal violet (CV), (b) methylene blue (MB), (c) rhodamine B (RhB), and (d) methyl orange (MO) on TS-1.


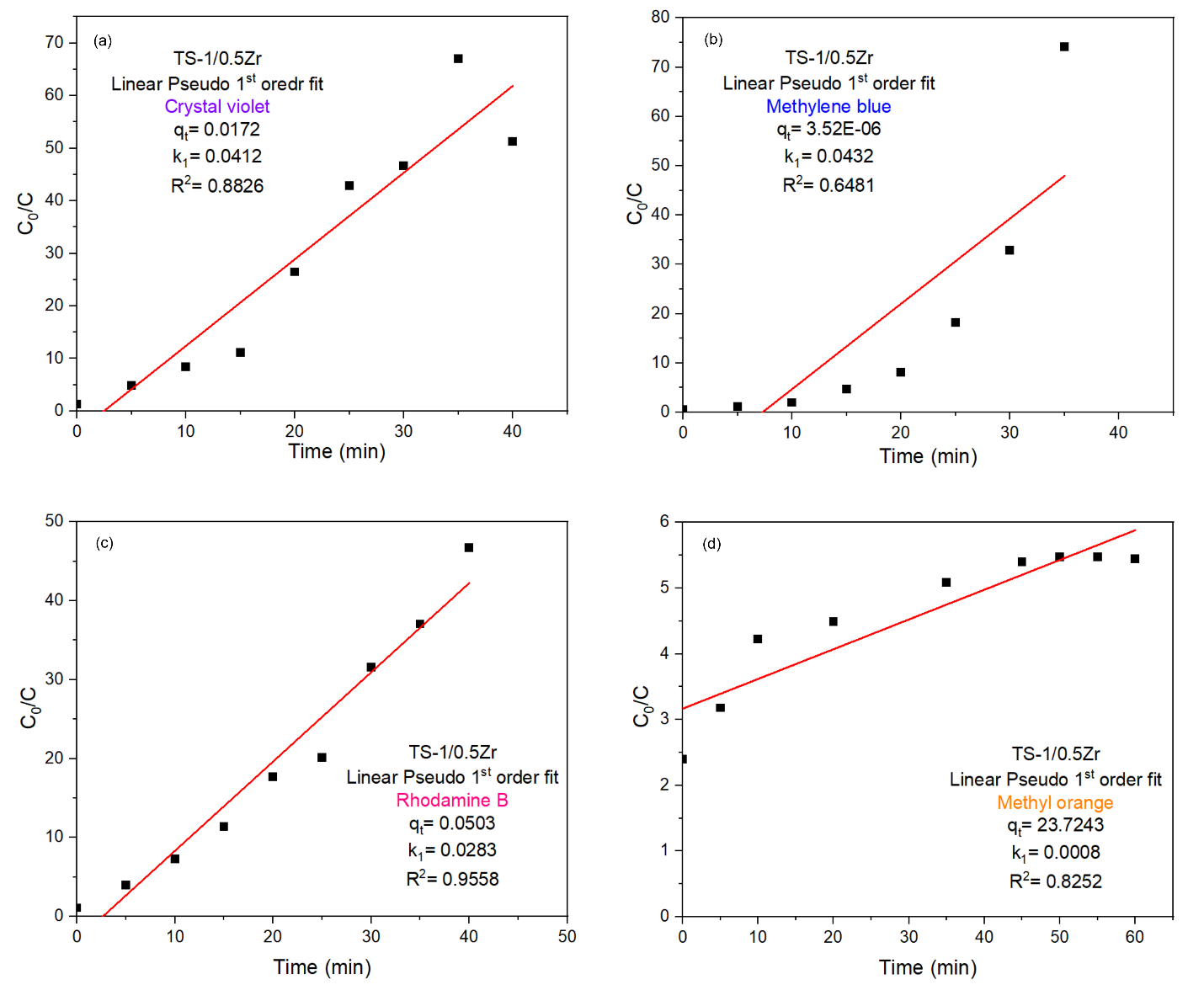


# Figure S8. Linear pseudo-first-order (PFO) kinetic model fits for the adsorption of (a) crystal violet (CV), (b) methylene blue (MB), (c) rhodamine B (RhB), and (d) methyl orange (MO) on TS-1/0.5Zr.


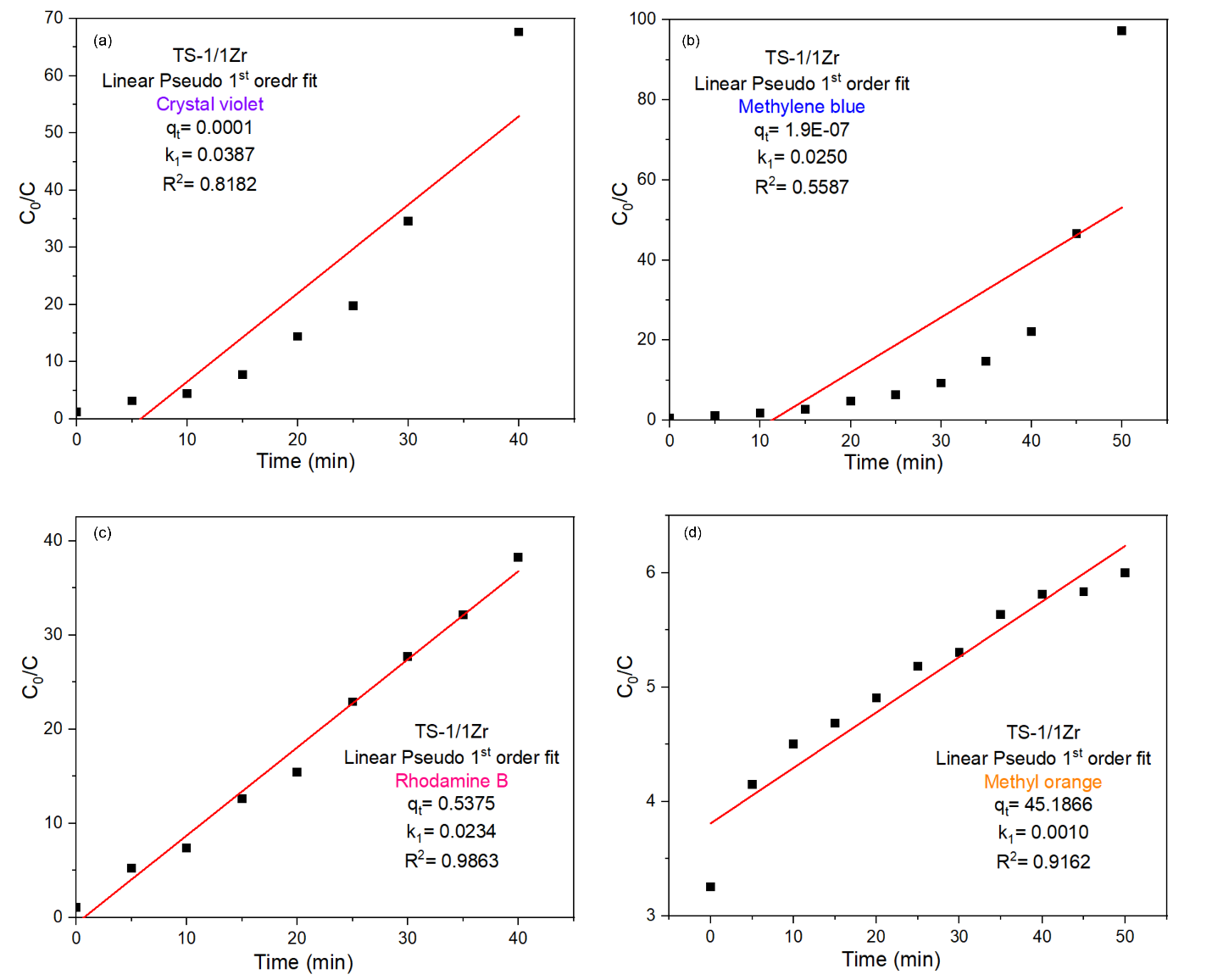


# Figure S9. Linear pseudo-first-order (PFO) kinetic model fits for the adsorption of (a) crystal violet (CV), (b) methylene blue (MB), (c) rhodamine B (RhB), and (d) methyl orange (MO) on TS-1/1Zr.


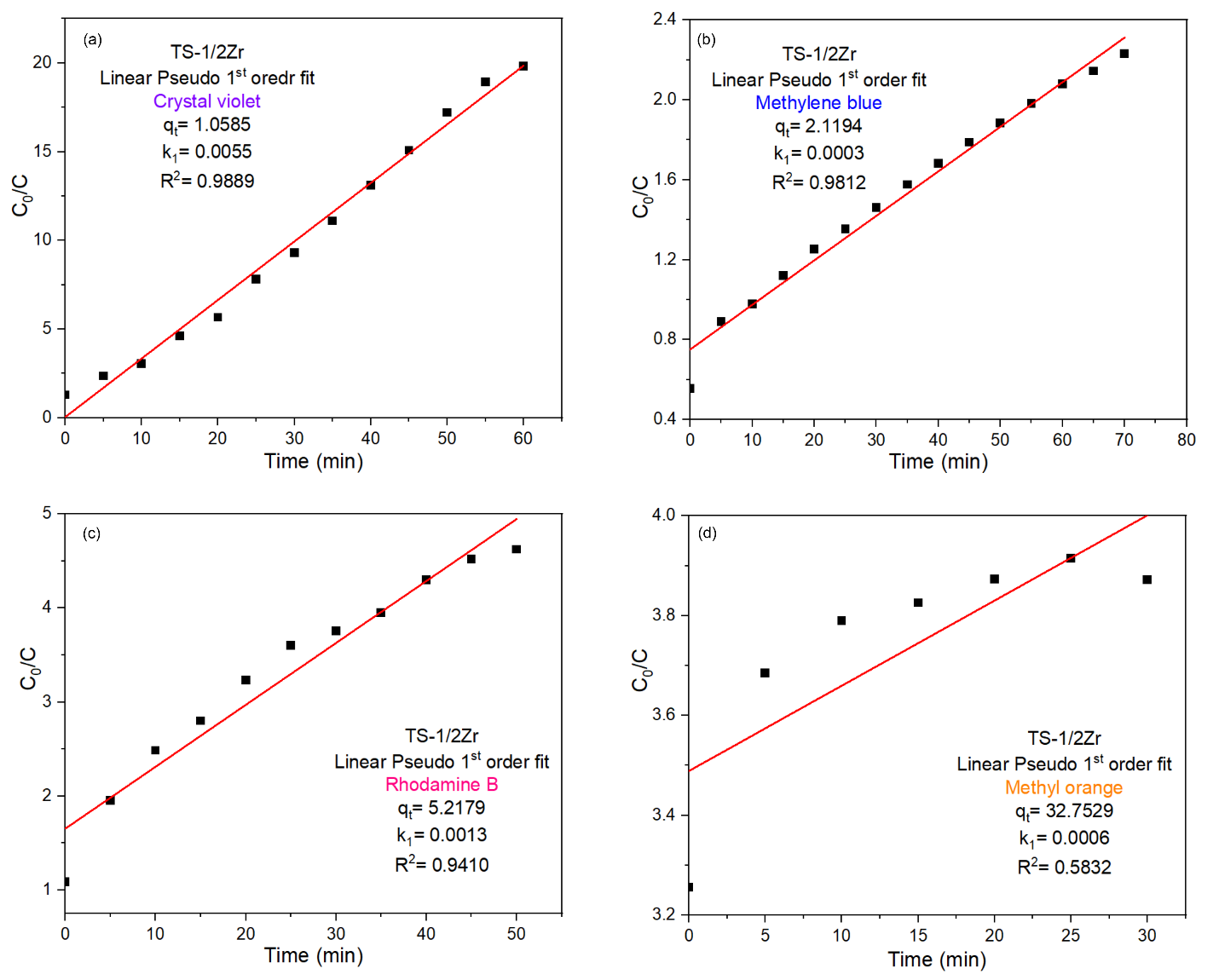


# Figure S10. Linear pseudo-first-order (PFO) kinetic model fits for the adsorption of (a) crystal violet (CV), (b) methylene blue (MB), (c) rhodamine B (RhB), and (d) methyl orange (MO) on TS-1/2Zr.


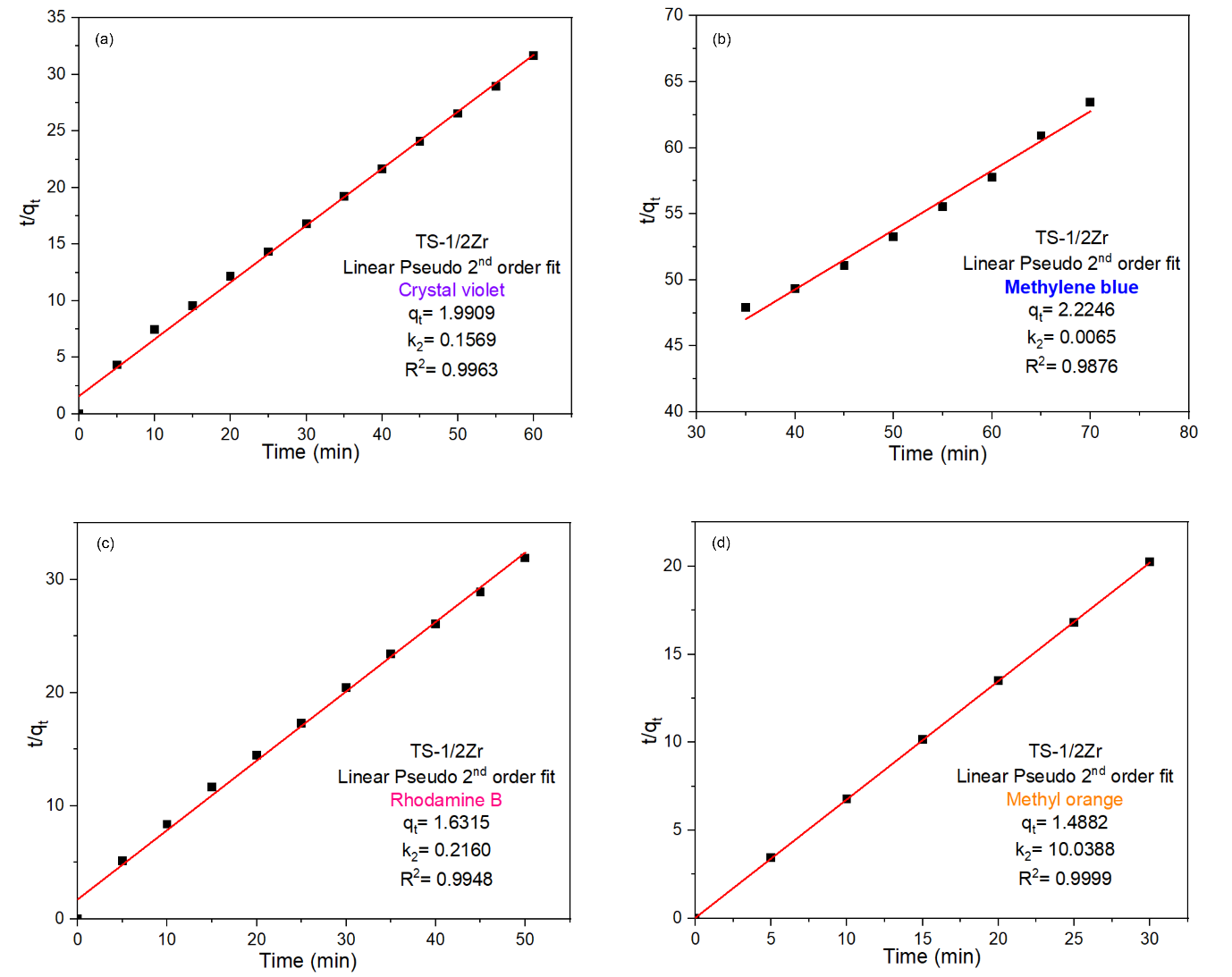


# Figure S11. Linear pseudo-second-order (PSO) kinetic model fits for the adsorption of (a) crystal violet (CV), (b) methylene blue (MB), (c) rhodamine B (RhB), and (d) methyl orange (MO) on TS-1/2Zr.


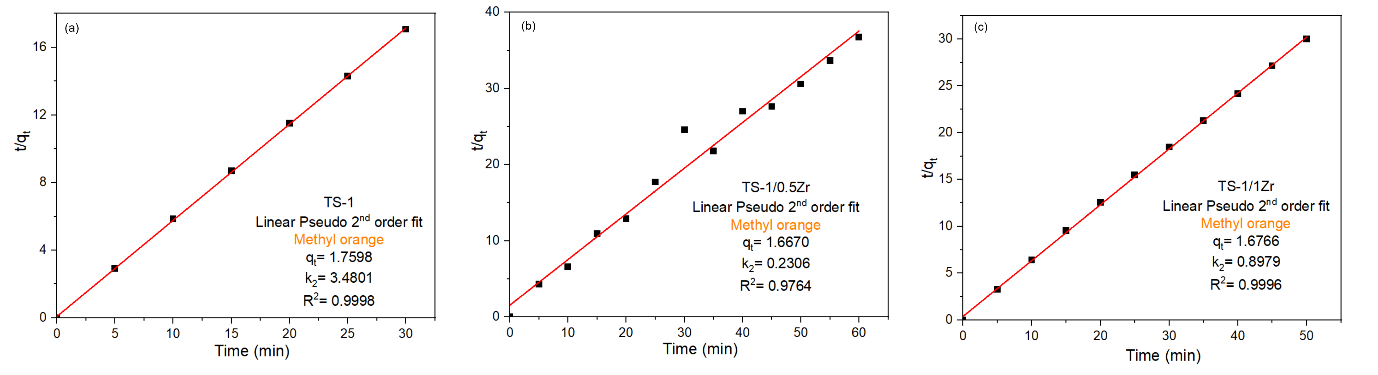


# Figure S12. Linear pseudo-second-order (PSO) kinetic model fits for the adsorption of methyl orange (MO) on (a) TS-1, (b) TS-1/0.5Zr, (c) TS-1/1Zr.


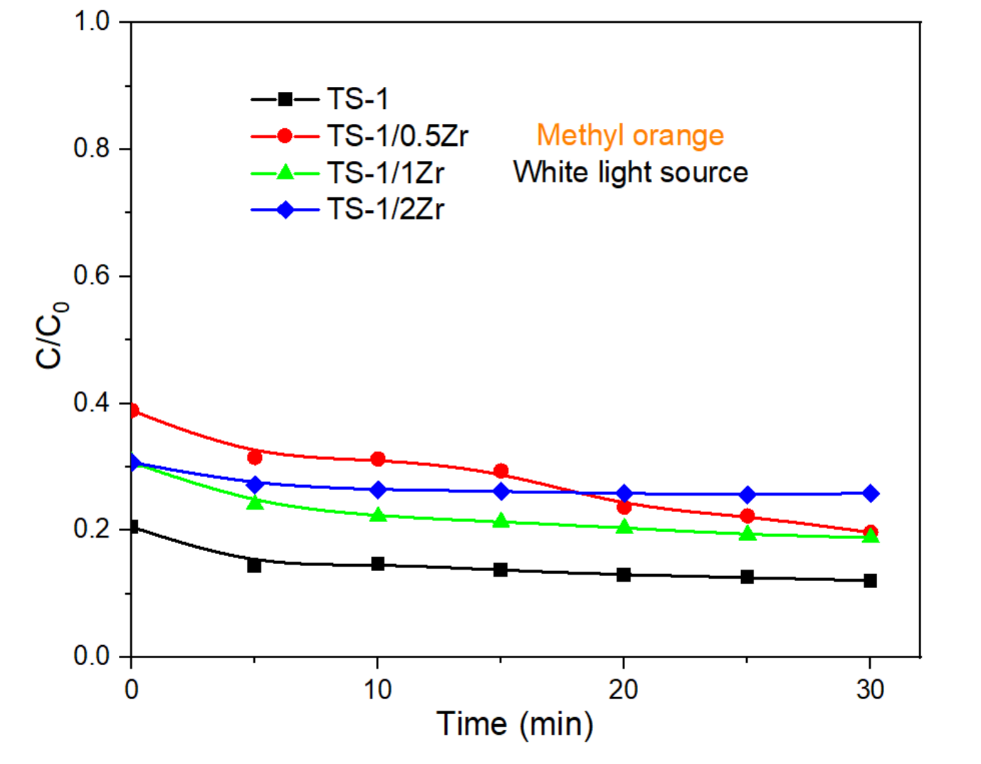


# Figure S13. Photodegradation of MO promoted by white light irradiation in the presence of TS-1 (black), TS-1/0.5Zr (red), TS-1/1Zr (green) and TS-1/2Zr (blue); the line connecting the dots is provided as a guide for the eye.


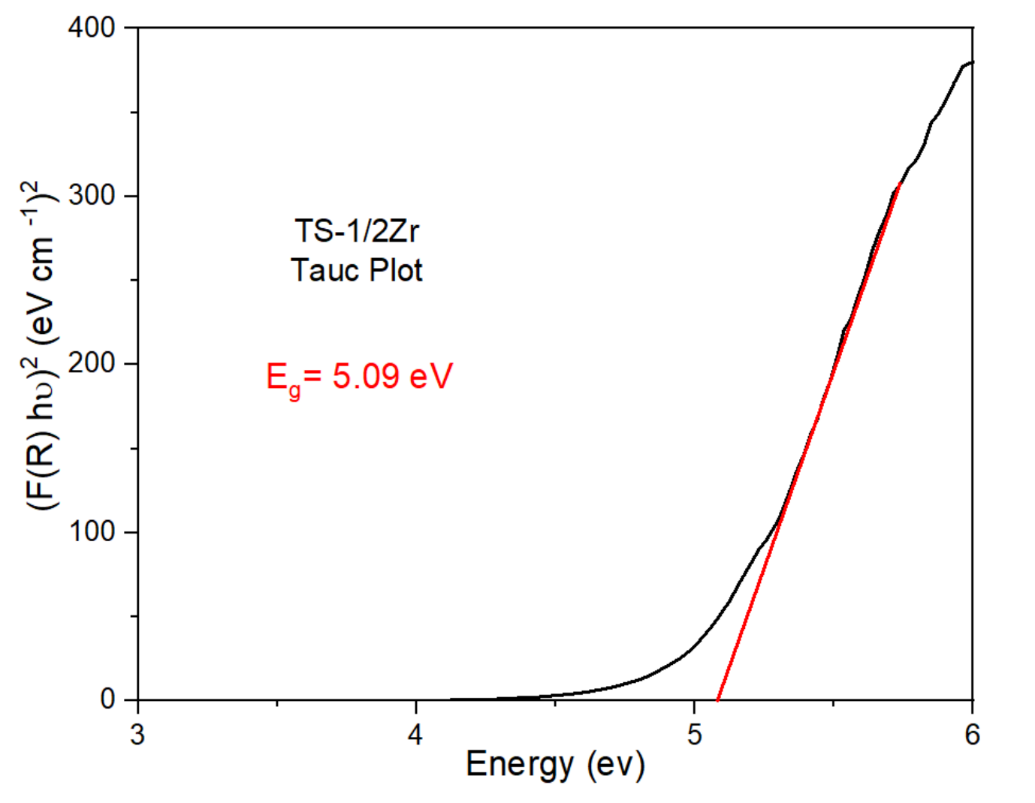


# Figure S14**.** Energy band gap for TS-1/2Zr samples obtained from Tauc plots.
